# Jaw1/LRMP increases Ca^2+^ influx upon GPCR stimulation with heterogeneous effect on the activity of each ITPR subtype

**DOI:** 10.1038/s41598-022-13620-4

**Published:** 2022-06-08

**Authors:** Wataru Okumura, Takuma Kozono, Hiroyuki Sato, Hitomi Matsui, Tsubasa Takagi, Takashi Tonozuka, Atsushi Nishikawa

**Affiliations:** 1 Department of Food and Energy Systems Science, Graduate School of Bio-Applications and Systems Engineering, Tokyo University of Agriculture and Technology, Tokyo, 184-8588 Japan; 2 Institute of Global Innovation Research, Tokyo University of Agriculture and Technology, Tokyo, 183-8509 Japan; 3 Cooperative Major in Advanced Health Science, Tokyo University of Agriculture and Technology, Tokyo, 184-8588 Japan; 4 Department of Applied Biological Chemistry, Graduate School of Agriculture, Tokyo University of Agriculture and Technology, Tokyo, 183-8509 Japan

**Keywords:** Ion channels, Cell biology, Molecular biology

## Abstract

Ca^2+^ influx upon G protein-coupled receptor (GPCR) stimulation is observed as a cytosolic Ca^2+^ concentration oscillation crucial to initiating downstream responses including cell proliferation, differentiation, and cell–cell communication. Although Jaw1 is known to interact with inositol 1,4,5-triphosphate receptor (ITPRs), Ca^2+^ channels on the endoplasmic reticulum, the function of Jaw1 in the Ca^2+^ dynamics with physiological stimulation remains unclear. In this study, using inducible Jaw1-expressing HEK293 cells, we showed that Jaw1 increases Ca^2+^ influx by GPCR stimulation via changing the Ca^2+^ influx oscillation pattern. Furthermore, we showed that Jaw1 increases the Ca^2+^ release activity of all ITPR subtypes in a subtly different manner. It is well known that the Ca^2+^ influx oscillation pattern varies from cell type to cell type, therefore these findings provide an insight into the relationship between the heterogeneous Ca^2+^ dynamics and the specific ITPR and Jaw1 expression patterns.

## Introduction

Intracellular Ca^2+^ dynamics are crucial to regulating diverse cellular processes, including cell proliferation, differentiation, and cell–cell communication^1–3^. Intracellular Ca^2+^ is stored in the endoplasmic reticulum (ER), and cell surface receptor-mediated extracellular stimuli trigger Ca^2+^ influx into the cytoplasm to initiate a physiological response^4^.

To date, the involvement of several basic Ca^2+^ channels on the ER membrane and cell surface has been described in stimuli-dependent Ca^2+^ dynamics regulation. Inositol 1,4,5-triphosphate receptors (ITPRs), Ca^2+^ channels on the ER membrane, release Ca^2+^ from the ER into the cytoplasm in an IP_3_-dependent manner when the IP_3_ is produced upon G protein-coupled receptor (GPCR)-related stimulation^5,6^. The Ca^2+^ efflux from the ER subsequently leads to Ca^2+^ depletion in the ER, triggering the activation of stromal-interaction molecule (STIM), a Ca^2+^ sensor protein on the ER^7,8^. The activated STIM interacts with Orai, a plasma membrane Ca^2+^ channel, resulting in Ca^2+^ influx from the extracellular source into the cytoplasm, called store-operated calcium entry (SOCE). The cytoplasmic Ca^2+^ is retrieved by sarco/endoplasmic reticulum Ca^2+^ ATPase (SERCA), a Ca^2+^ pump on the ER, resulting in Ca^2+^ replenishment in the ER^9^. This Ca^2+^ dynamics series is repeated and observed as a cytosolic Ca^2+^ concentration ([Ca^2+^]_c_) oscillation. Interestingly, recent studies revealed that the oscillation, as well as the cytoplasmic Ca^2+^ influx level, is important for Ca^2+^-dependent protein activation^10,11^.

Jaw1, also known as lymphoid restricted membrane protein (LRMP), is a type II integral membrane protein with specific expression in immune cells, taste cells, pancreatic acinar cells, and small intestinal tuft cells^12–15^. We previously reported that Jaw1 is localized on the ER and the outer nuclear membrane, and it is associated with nuclear shape maintenance^16^. Furthermore, Jaw1 was recently reported to interact with all ITPR subtypes via its coiled-coil domain, and the deletion of Jaw1 in mouse embryonic fibroblast (MEF) reduces Ca^2+^ influx into the cytoplasm upon ionomycin stimulation, a reagent that increases cell membrane ion permeability^13^. However, whether and how Jaw1 is involved in the calcium dynamics under physiological conditions, such as GPCR stimulation-related aspects, remains unclear. Furthermore, physiological differences have been reported among ITPR1–3, three ITPR isoforms, such as IP_3_ affinity, Ca^2+^ release activity, and in vivo expression patterns^17–20^, but it remains elusive whether Jaw1 affects the ITPR activities subtype-specifically.

In this study, we showed that Jaw1 increases cytoplasmic Ca^2+^ influx upon GPCR stimulation, leading to changes in the Ca^2+^ influx pattern. Furthermore, our data indicated that Jaw1 interacts with all ITPR subtypes, increasing their Ca^2+^ release activity with subtle differences among each subtype. These results contribute to our understanding of the physiological significance of the heterogeneous Ca^2+^ dynamics among cell types and the involvement of Jaw1, as an ITPR regulator, in the uniqueness of Ca^2+^ dynamics in tissues or cells.

## Results

### Jaw1 increases the Ca^2+^ influx in an expression level-dependent manner

To uncover how Jaw1 affects Ca^2+^ dynamics upon physiological stimulation, we created HEK293 Flp-In T-REx Jaw1 inducible-expressed cells with doxycycline (Dox) treatment-inducible Jaw1 expression. As previously reported, endogenous Jaw1 is expressed in HEK293 cells at a low level^21^. First, we generated HEK293 Flp-In T-REx Jaw1 KO cells (Jaw1 KO cells) by CRISPR/Cas9 (see Supplementary Fig. S1A online), although we could not detect the specific Jaw1 band by western blotting due to the low expression level (see Supplementary Fig. S1B online). We validated by Sanger sequencing a stop codon insertion due to the frameshift upstream of the coiled-coil domain, an essential Jaw1-ITPR interaction domain. The genes of Jaw1 or Jaw1 Δcoil (lacking the coiled-coil domain) were then introduced into Jaw1 KO cells, and their inducible expressions (hereafter Jaw1 IE and Jaw1 Δcoil cells, respectively) were validated by western blotting and immunostaining (see Supplementary Fig. S1C–E online). We also confirmed that the ITPR expression levels were not altered in any cell (see Supplementary Fig. S1F–I online).

**Figure 1:**
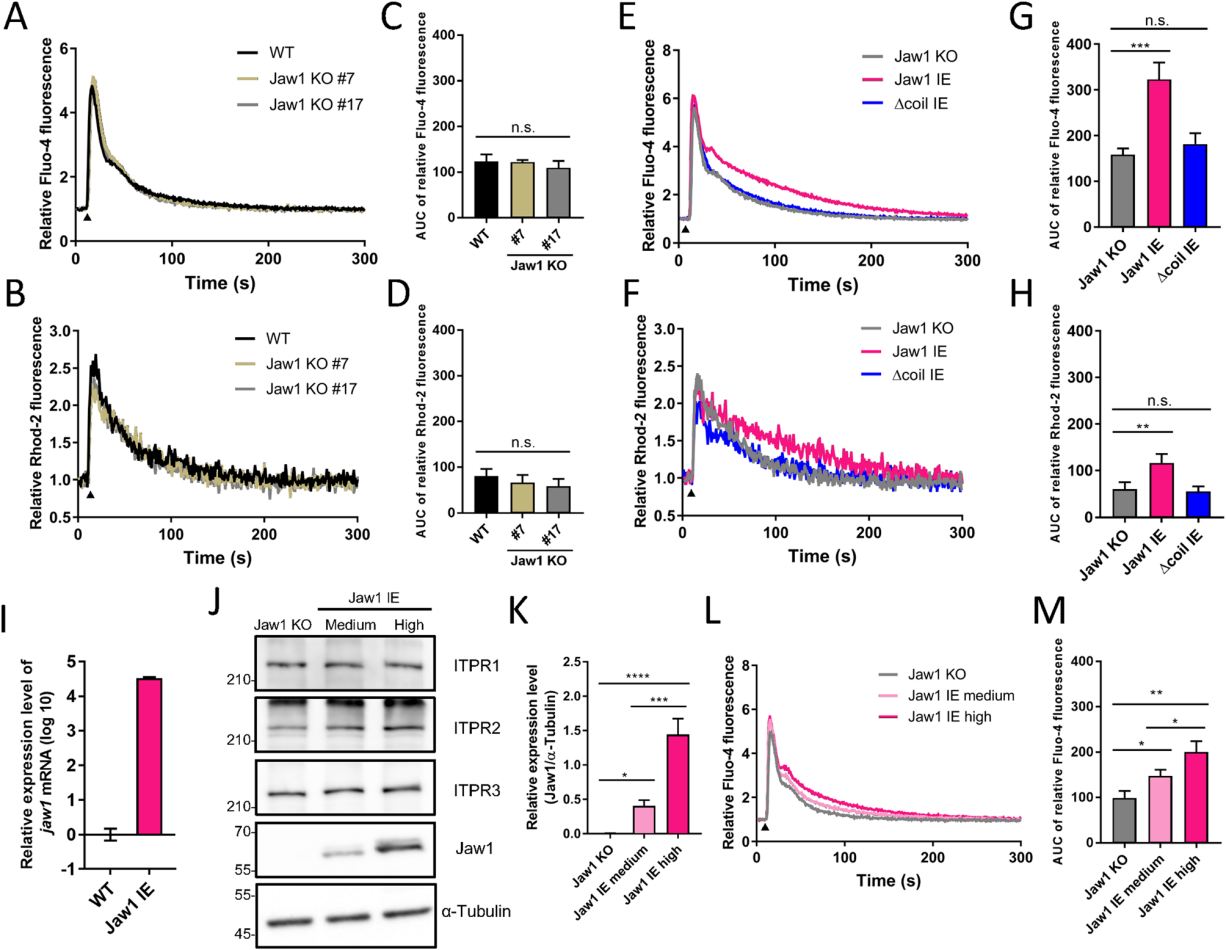
Jaw1 increases the Ca^2+^ influx depending on its own expression level. (**A**,**B**,**E**,**F**,**L**) Mean curves of relative Fluo-4 (**A**,**E**,**L**) or Rhod-2 (**B**,**F**) intensity upon 100 µM ATP stimulation measured using a plate reader. WT, Jaw1 KO #7, and Jaw1 KO #17 cells (**A**,**B**); Jaw1 KO, Jaw1 IE, and Δcoil IE cells (**E**,**F**); or Jaw1 KO, Jaw1 IE medium, and Jaw1 IE high cells (**L**). The closed triangles indicate the time point of 100 µM ATP solution supplementation. (**C**,**D**,**G**,**H**,**M**) AUC in (**A**), (**B**), (**E**), (**F**), and (**L**) are shown in (**C**), (**D**), (**G**), (**H**), and (**M**), respectively. n = 3. (**I**) Relative expression level of *jaw1* mRNA to *gapdh* in WT and Jaw1 IE cells measured by RT-qPCR. N = 3. (**J**) Jaw1 KO and Jaw1 IE cells treated with 0.25 or 200 ng/mL of Dox and subjected to western blotting. Images used for western blots are shown in Supplementary Fig. S4 online. (**K**) The relative protein expression level of Jaw1 with α-Tubulin in (**J**). n = 3. The error bar shows ± S.D.; n.s., non-significant; *p < 0.05; **p < 0.01; ***p < 0.001; ****p < 0.0001, Tukey–Kramer’s *t*-test.

To evaluate the Ca^2+^ flux, the above-described cells were loaded with Fluo-4 and Rhod-2, cytoplasmic and mitochondrial Ca^2+^ indicators, respectively. The Ca^2+^ flux of each cell in response to 100 μM ATP, a GPCR stimulant, was then measured using a plate reader. The Fluo-4 and Rhod-2 kinetic curves almost overlapped among the WT cells and two Jaw1 KO cell clones (Fig. 1A,B). Similarly, the area under the curve (AUC), indicating the total cytosolic and mitochondrial Ca^2+^ influx, was also comparable (Fig. 1C,D). However, the kinetic curves of both Fluo-4 and Rhod-2 in Jaw1 IE cells indicated high-intensity maintenance compared to those in Jaw1 KO cells (Fig. 1E,F). Similarly, the total Ca^2+^ influx into the cytosol and mitochondria in Jaw1 IE cells significantly increased by approximately 35% and 10% in Fluo-4 and Rhod-2, respectively, compared to those in KO cells (Fig. 1G,H). In contrast, in the Δcoil IE cells, the kinetic curves and the AUC were comparable with those in the Jaw1 KO cells (Fig. 1E–H). Furthermore, we tested the carbachol to stimulate the muscarinic acetylcholine receptor3 in order to confirm that the augmentative effect of Jaw1 on the Ca^2+^ influx is mediated with GPCR. Similar to the result of ATP stimulation, the kinetic curve in Jaw1 IE cells indicated high-intensity maintenance and AUC significantly increased compared to those in KO cells (see Supplementary Fig. S2 online). Therefore, these results indicated that Jaw1 expression increases the Ca^2+^ influx into the cytoplasm and mitochondria upon GPCR stimulation via its interaction with ITPRs.

Interestingly, the Ca^2+^ kinetics was not altered in WT and Jaw1 KO but increased only in Jaw1 IE cells. Moreover, the RT-qPCR results showed that the *jaw1* mRNA expression level in WT cells was approximately 30,000-fold lower than that in Jaw1 IE cells (Fig. 1I). These results give us the idea that Jaw1 increases the Ca^2+^ influx depending on its own expression level. Therefore, through treatments with different Dox concentrations (0.25 and 200 ng/mL, respectively), we prepared cells expressing Jaw1 at medium and high levels (Fig. 1J,K). Expectedly, the cytoplasmic Ca^2+^ influx increased in the order of Jaw1 IE medium and high cells (Fig. 1L,M). In summary, these results indicated that Jaw1 increases Ca^2+^ influx upon the GPCR stimulation in an expression level-dependent manner.

### Jaw1 increases Ca^2+^ influx by changing the influx pattern

**Figure 2:**
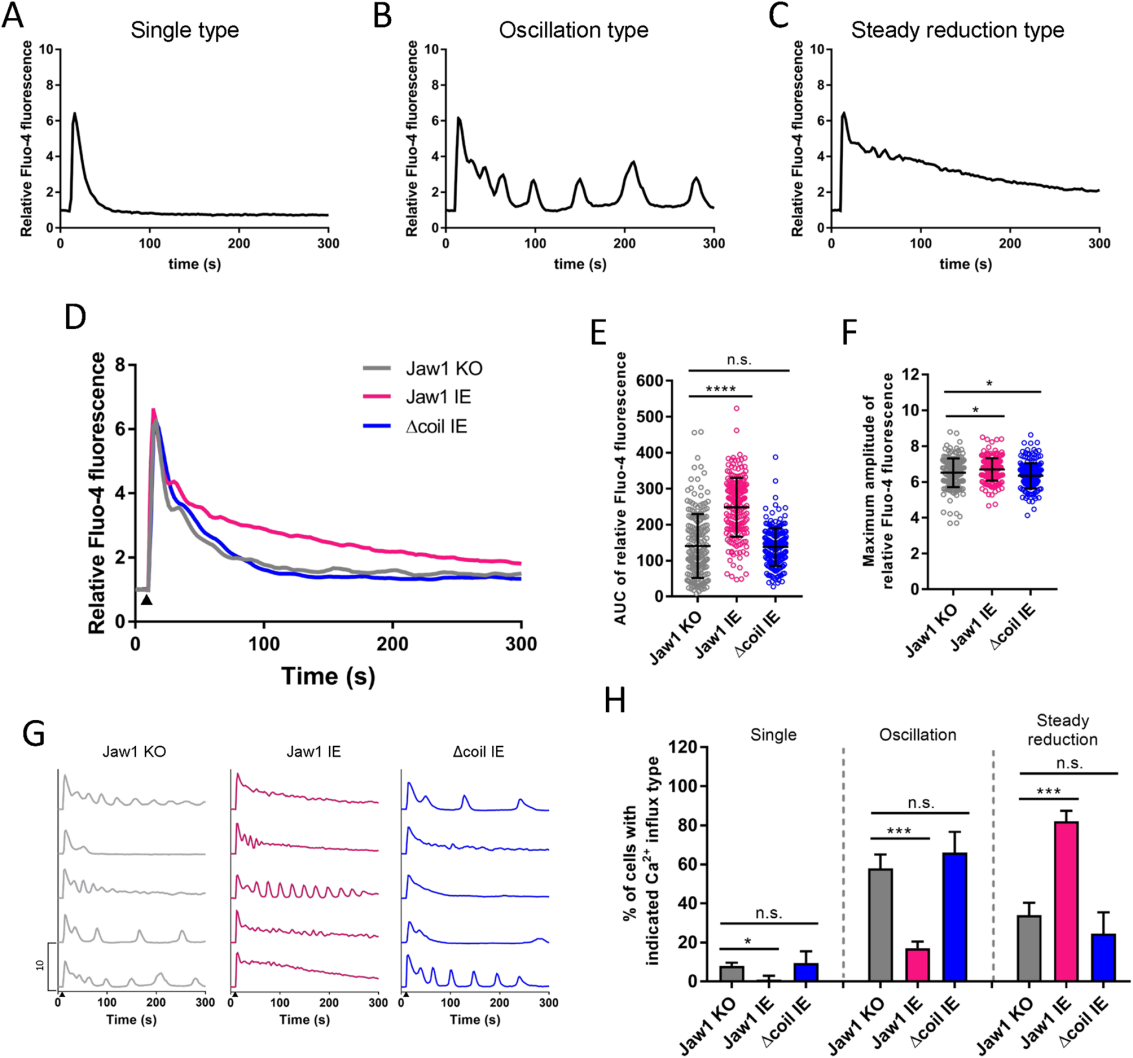
Jaw1 increases Ca^2+^ influx by changing the Ca^2+^ influx pattern. (**A**,**B**,**C**) Representative relative intensity traces upon 100 µM ATP stimulation: (**A**) single, (**B**) oscillation, and (**C**) steady reduction types. (**D**,**G**) Mean (**D**) or five representative (**G**) curves of the relative intensity in Jaw1 KO, Jaw1 IE, and Δcoil IE cells. The closed triangles indicate the time point of 100 µM ATP solution supplementation. n = 200. (**E**,**F**) AUC (**E**) and maximum amplitude (**F**) of the relative intensity from (**D**). (**H**) Ca^2+^ influx type classification in Jaw1 KO, Jaw1 IE, and Δcoil cells. Four independent experiments (n = 50). The error bars show ± S.D.; n.s., non-significant; *p < 0.05; ***p < 0.001; ****p < 0.0001, Tukey–Kramer’s *t*-test.

The GPCR stimulation-induced cytoplasmic Ca^2+^ influx pattern reportedly differs in each cell, even in the same cell line, and can be classified into three major types^22^. The first is a cell with a “single type” pattern, in which the Ca^2+^ influx occurs only the first time immediately after the GPCR stimulation with no Ca^2+^ influx thereafter (Fig. 2A). The second is a cell with an “oscillation type” pattern, in which the first Ca^2+^ influx is followed by a subsequent Ca^2+^ influx after GPCR stimulation (Fig. 2B). The third is a cell with a “steady reduction type” pattern, in which the [Ca^2+^]_c_ continuously remains high after GPCR stimulation (Fig. 2C). Therefore, we investigated whether Jaw1 expression could change the Ca^2+^ influx pattern and performed single-cell Ca^2+^ imaging using fluorescence microscopy. Here, we performed time-lapse imaging of the relative Fluo-4 fluorescence imaging for 5 min upon ATP stimulation in Jaw1 KO, Jaw1 IE, and Δcoil IE cells. The mean curve and the AUC in Jaw1 IE cells increased, and the [Ca^2+^]_c_ in Δcoil IE cells maintained the same trend as those in Jaw1 KO cells (Fig. 2D,E), consistent with the data measured using the plate reader (Fig. 1E,G). The maximum amplitude, indicating the maximum [Ca^2+^]_c_ after the stimulation, displayed a subtle difference (~ 5%) among the cell lines (Fig. 2F). Subsequently, we classified the Ca^2+^ influx pattern in each cell as described above (Fig. 2G,H). Interestingly, in Jaw1 KO cells, we detected 8, 58, and 34% of the single, oscillation, and steady reduction types, respectively, compared to reductions to 1% and 17% and a significant increase to 82% of the single, oscillation, and steady reduction types, respectively, in Jaw1 IE cells. Importantly, the composition ratio in Δcoil IE cells was almost the same as that in Jaw1 KO cells. These data indicate that Jaw1 changes the Ca^2+^ influx pattern augmentatively and increases the cytoplasmic Ca^2+^ influx frequency upon GPCR stimulation via interaction with ITPRs, resulting in continuously high [Ca^2+^]_c_ on average.

**Figure 3:**
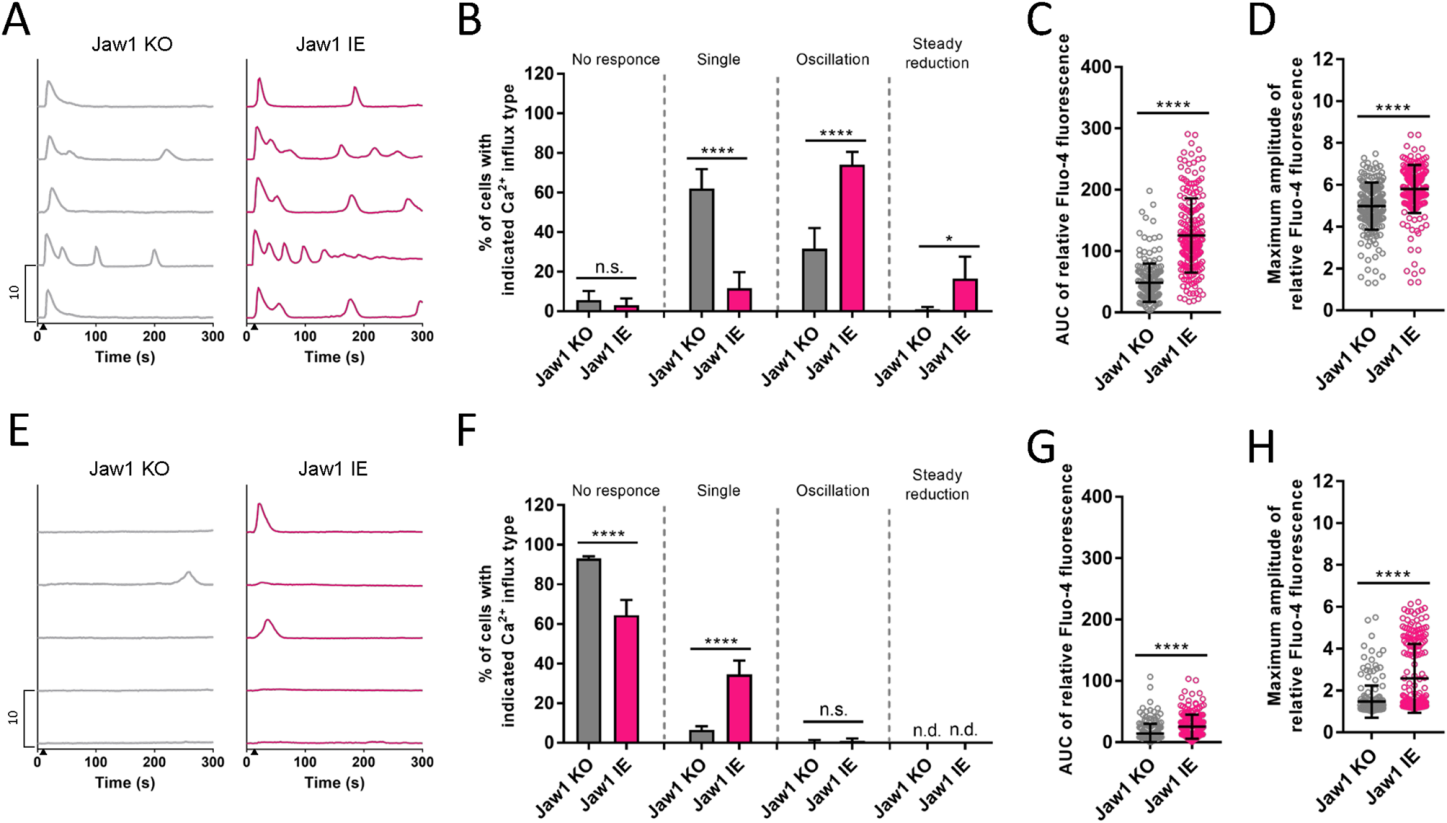
The Jaw1 augmentative effect on the Ca^2+^ influx is maintained even under weak GPCR stimulation. (**A**,**E**) Five representative relative Fluo-4 fluorescence intensity responses to the stimulation with 1 µM (**A**) or 0.3 µM (**E**) ATP out of 200 cells. The closed triangles show the time point of ATP stimulation. (**B**,**F**) Ca^2+^ influx type classification in each cell line upon stimulation with 1 µM (**B**) or 0.3 µM (**F**) ATP. The ratios were calculated based on the average of four independent wells. (**C**,**D**,**G**,**H**) Graph representing the AUC (**C**,**G**) and maximum amplitude (**D**,**H**) of the relative Fluo-4 fluorescence intensity in each cell line upon stimulation with 1 µM (**C**,**D**) or 0.3 µM (**G**,**H**) ATP in 200 cells. The error bar shows ± S.D.; ****p < 0.0001. Student’s *t*-test.

To further investigate whether the augmentative Jaw1 effect on Ca^2+^ influx is maintained even upon weak GPCR stimulation, similar experiments were performed at lower ATP concentrations of 1 and 0.3 µM. Here, we defined a new cell type of “no response” since no Ca^2+^ influx could be observed upon the weak ATP stimulation. In the case of the 1 µM ATP stimulation, fewer Jaw1 IE cells were of the single type and more of the oscillation type than Jaw1 KO cells (Fig. 3A,B). In the case of the 0.3 µM ATP stimulation, most Jaw1 KO cells were of the no response type, while 35% of the Jaw1 IE cells responded (Fig. 3E,F). In addition, the total Ca^2+^ influx and maximum amplitude in the Jaw1 IE cells also increased significantly under both conditions (Fig. 3C,D,G,H). In summary, Jaw1 changes the Ca^2+^ influx pattern augmentatively even upon weak GPCR stimulation.

### Jaw1 accelerates Ca^2+^ efflux from the ER

**Figure 4:**
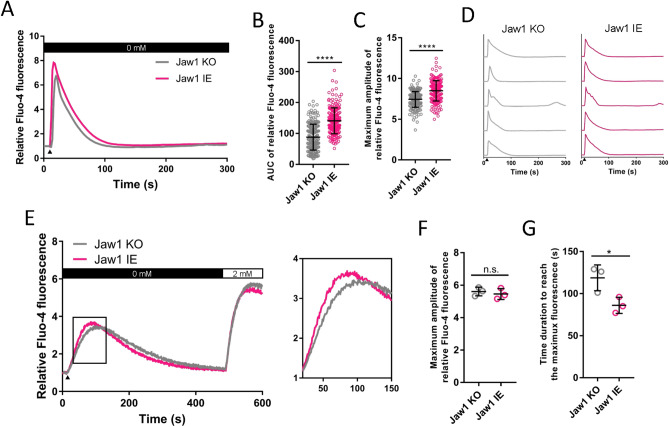
Jaw1 accelerates Ca^2+^ efflux from ER**.** (**A**,**D**) Mean (**A**) or five representative (**D**) curves of the relative intensity upon 100 µM ATP stimulation under extracellular Ca^2+^ free condition in Jaw1 KO and Jaw1 IE cells. n = 200. The closed triangle shows the time point of 100 µM ATP solution supplementation. (**B**,**C**) AUC (**B**) or maximum amplitude (**C**) of the relative intensity from (**A**). (**E**) Mean curves of the relative intensity upon Ca^2+^ depletion in the ER by 2 µM thapsigargin supplementation in 0 mM Ca^2+^ and replenishment with 2 mM Ca^2+^. The closed triangle shows the time point of 2 µM thapsigargin solution supplementation. The area surrounded with a black box is enlarged in the right. n = 3. (**F**,**G**) Maximum amplitude in SOCE (**F**) and time duration to reach the maximum amplitude in the Ca^2+^ leakage (**G**) from (**E**). The error bar shows ± S.D.; n.s., non-significant; *p < 0.05; ****p < 0.0001, Student’s *t*-test.

Our data using the Jaw1 mutant (Δcoil) suggest that the Jaw1–ITPR interaction on the ER increases the Ca^2+^ efflux from the ER into the cytoplasm. However, the GPCR stimulation-induced Ca^2+^ influx does not only originate from the ER but also extracellular sources such as SOCE via the STIM-Orai complex. Therefore, to determine whether the Jaw1-mediated increased Ca^2+^ influx is of an ER or extracellular source upon GPCR stimulation, we observed cytosolic Ca^2+^ influx in Jaw1 KO and Jaw1 IE cells upon ATP stimulation in the absence of extracellular Ca^2+^. The results showed that the mean curve in Jaw1 IE cells was maintained higher than that of Jaw1 KO cells up to approximately 100 s even in the absence of extracellular Ca^2+^ (Fig. 4A). The total Ca^2+^ influx and maximum amplitude were also significantly higher in Jaw1 IE cells than in Jaw1 KO cells (Fig. 4B,C), indicating that Jaw1 increased the Ca^2+^ efflux from the ER. However, the classified Ca^2+^ influx pattern in each cell line showed that almost all cells in both the Jaw1 KO and Jaw1 IE lines were of the single type (Fig. 4D). Ca^2+^ influx from an extracellular source is reportedly required to produce the following oscillation^23^. Our results thus suggest that Ca^2+^ replenishment from the extracellular source is required prior to the Jaw1 expression-mediated augmentative Ca^2+^ oscillation. Furthermore, to examine whether Jaw1 was involved with SOCE, Jaw1 KO and Jaw1 IE cells were treated with thapsigargin, a Ca^2+^ transport protein inhibitor on the ER membrane SERCA, to deplete Ca^2+^ in the ER and activate STIM in the absence of extracellular Ca^2+^. The extracellular Ca^2+^ was then replenished to induce SOCE. The replenishment with 2 mM Ca^2+^ did not change the curve and maximum values in Jaw1 KO and Jaw1 IE cells, indicating that Jaw1 did not change the SOCE activity (Fig. 4E,F). However, interestingly, Ca^2+^ leakage from the ER during the thapsigargin treatment was significantly faster in the Jaw1 IE cells than in the Jaw1 KO cells (Fig. 4E,G), indicating that Jaw1 increases the Ca^2+^ release from the ER even in a steady state without GPCR stimulation. In summary, Jaw1 increases the cytoplasmic Ca^2+^ influx by accelerating the Ca^2+^ efflux from the ER.

### Jaw1 increases the activity of all ITPR subtypes in subtly different ways

**Figure 5:**
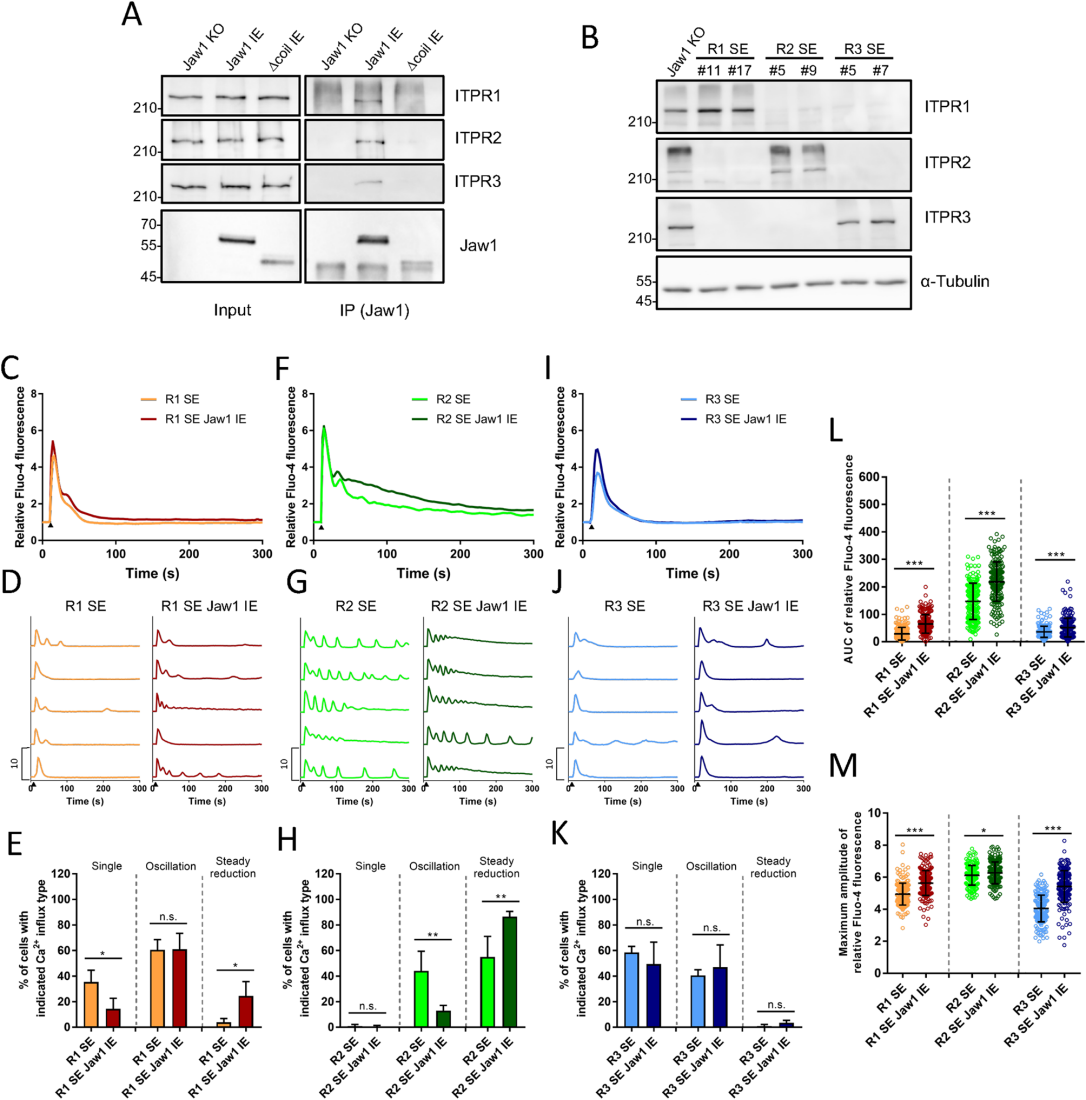
Jaw1 increases the activity of all ITPR subtypes with subtle differences. (**A**) Jaw1 KO, Jaw1 IE, and Δcoil IE cell lysates were subjected to co-immunoprecipitation followed by western blotting. (**B**) Jaw1 KO, R1 SE #11 or #17, R2 SE #5 or #9, and R3 SE #5 or #7 cells were subjected to western blotting. Images used for western blots of (**A**) and (**B**) are shown in Supplementary Fig. S5, S6 online. (**C**,**D**,**F**,**G**,**I**,**J**) Mean (**C**,**F**,**I**) or five representative (**D**,**G**,**J**) curves of the relative intensity in R1 SE, R2 SE, and R3 SE with or without Jaw1 IE cells. Closed triangles indicate the time point of 100 µM ATP solution supplementation. n = 200. (**E**,**H**,**K**) Classification of the Ca^2+^ influx type in each cell line. Four independent experiments (n = 50). (**L**,**M**) AUC (**L**) or maximum amplitude (**M**) of the relative intensity from (**C**), (**F**), and (**I**). The error bar shows ± S.D.; n.s., non-significant; *p < 0.05; **p < 0.01; ***p < 0.001.

So far, three ITPR subtypes, ITPR1–3, have been reported with functional differences among them^17,18,24^. To confirm whether Jaw1 interacts with all ITPR subtypes, we first prepared Jaw1 KO, Jaw1 IE, and Δcoil IE cell lysates and performed co-immunoprecipitation. The results showed that all ITPR subtypes co-immunoprecipitated with Jaw1, but none with Δcoil, suggesting that Jaw1 interacts with all subtypes via its coiled-coil domain (Fig. 5A).

Furthermore, to investigate whether the augmentative effect of Jaw1 on the Ca^2+^ flux would be subtype-specific, we prepared Jaw1 KO ITPR single-expressing cells (R1/R2/R3 SE cells), expressing exclusively a single subtype of each ITPR (Fig. 5B). The Jaw1 gene was then introduced into these cells and the inducible Jaw1 expression (hereafter referred to as R1/R2/R3 SE Jaw1 IE cells) was validated by western blotting and immunostainings (see Supplementary Fig. S3A–D online). In addition, the Jaw1 expression did not affect the expression level of any ITPR subtype (see Supplementary Fig. S3E–G online). In this context, each cell line was subjected to time-lapse Ca^2+^ imaging upon ATP stimulation. In the R1 SE Jaw1 IE cells, the mean curve showed that a higher Ca^2+^ influx state could be maintained, the single type decreased from 36 to 15%, and the steady reduction type increased from 4 to 25%, compared to the R1 SE cells (Fig. 5C–E). Similarly, in the R2 SE Jaw1 IE cells, the mean curve showed a higher Ca^2+^ influx state, the oscillation type decreased from 44 to 13%, and the steady reduction type increased from 55 to 87%, compared to the R2 SE cells (Fig. 5F–H). Interestingly, in the case of R3 SE, the composition ratio did not change between the Jaw1 KO and Jaw1 IE cells (Fig. 5I–K). Although the AUC was significantly higher in all subtype SE cells when examining the expression of Jaw1, the increase is much more in R1 and R2 SE cells than that of R3 SE cells, consistent with the changes in the composition ratio (Fig. 5L). On the other hand, the maximum amplitude increased more in R3 SE cells than that of R1 and R2 SE cells. In summary, these results suggest that Jaw1 has an augmentative effect on the Ca^2+^ release activity of all ITPR subtypes with subtle differences among the ITPR subtypes.

## Discussion

**Figure 6:**
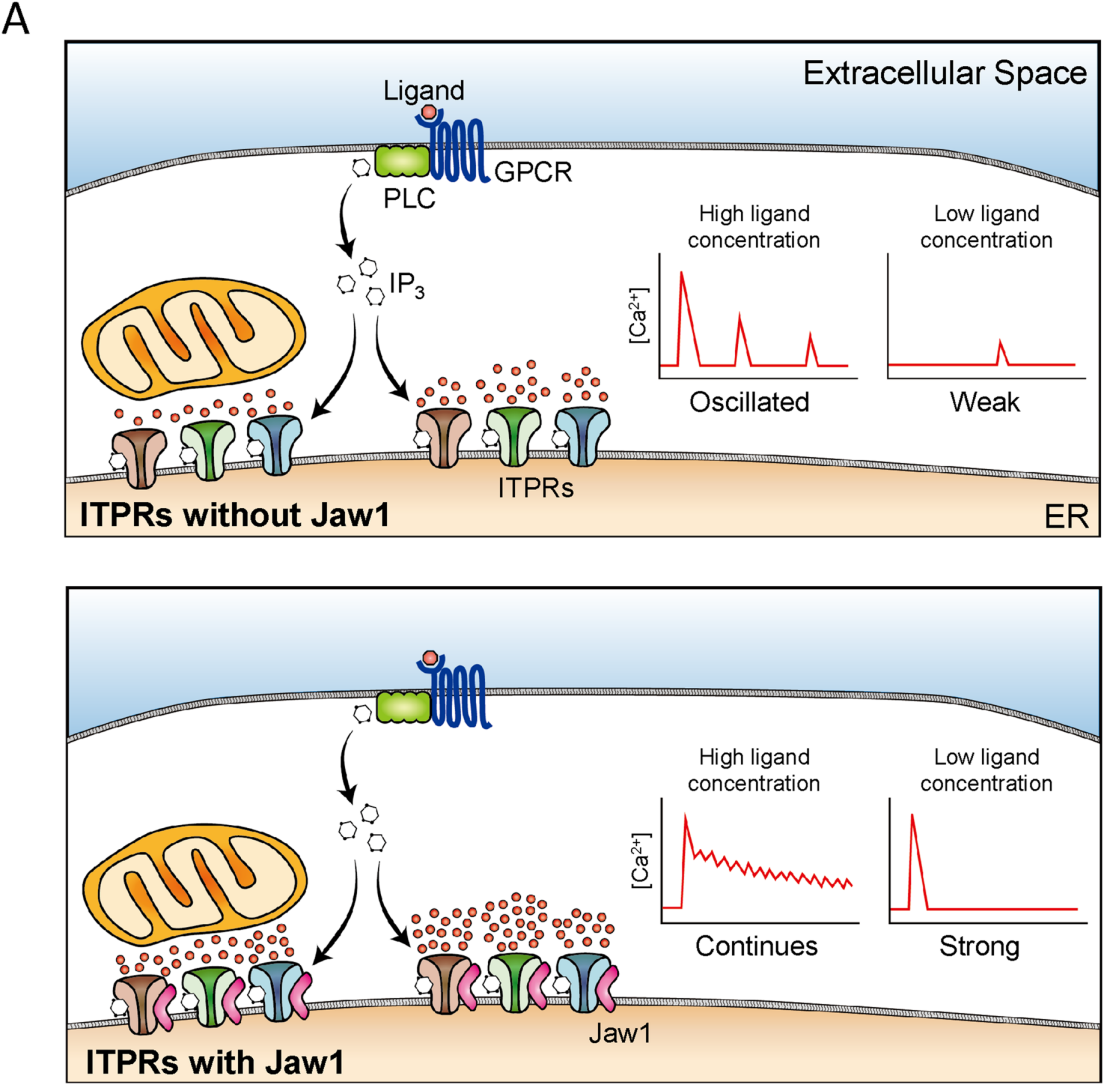
A model of the Jaw1 function as a regulator of the ITPRs activity upon GPCR stimulation. (**A**) Jaw1 promotes Ca^2+^ release activity of ITPRs and increases Ca^2+^ influx into cytoplasm and mitochondria upon GPCR stimulation. Jaw1 changes the Ca^2+^ influx pattern from oscillated response to continuous response in high GPCR stimulation, and from weak response to strong response in low GPCR stimulation.

In this study, we uncovered that Jaw1 increases the cytoplasmic and mitochondrial Ca^2+^ influx upon GPCR stimulation via interacting with ITPRs depending on its expression level. Importantly, single-cell imaging of the Ca^2+^ influx revealed that Jaw1 expression exerts the augmentative changes in the Ca^2+^ influx pattern in each cell. Our data also indicated that this increased cytoplasmic Ca^2+^ influx upon Jaw1 expression originates from the Ca^2+^ released from the ER and not the extracellular Ca^2+^ influx. Furthermore, Jaw1 increased the activity of all ITPR subtypes with subtle differences among subtypes (Fig. 6).

Interestingly, inositol 1,4,5-triphosphate receptor associate 1 (IRAG1), a Jaw1 homolog, is also known to interact with ITPRs but represses their activity unlike Jaw1^25,26^. The effect of IRAG1 on the ITPRs is regulated phosphorylation-dependently at S678, located just below the coiled-coil domain^27^. Similarly, a previous proteomic analysis has shown that Jaw1 also undergoes phosphorylation at S363, located right below the coiled-coil domain^28^, suggesting that the effect of Jaw1 on the ITPRs is also regulated by phosphorylation. Furthermore, the homology between IRAG1 and Jaw1 is less than 30%, and IRAG1 displays an extra sequence of approximately 200 amino acids on its N-terminal side in contrast to Jaw1^29^. Therefore, further studies using Jaw1 and IRAG1 phosphomimetic and domain-switching mutants could clarify how Jaw1 regulates the activity of ITPRs.

The cytoplasmic Ca^2+^ influx activates the Ca^2+^ adaptor proteins and enzymes in the cytoplasm, which transmit various signals^4^. Importantly, recent studies revealed that the oscillation, as well as the cytoplasmic Ca^2+^ influx level, is important for downstream protein activation^10,22,30^, suggesting that the oscillation-mediated duration of high [Ca^2+^]_c_ above a certain threshold could be a key factor to induce the physiological responses. Indeed, several cellular events, such as secretion and gene expression, reportedly take a few minutes to initiate a response after the stimulation^31^. Of interest, as shown in Figs. 2 and 3A–D, the data of single cell Ca^2+^ imaging in Jaw1 IE cells demonstrated that each kinetic curve is more oscillated than that of Jaw1 KO cells. Particularly, the curve for steady reduction type showed a trend of being highly oscillated, although each ratio was depending on the concentration of ligand as well as the expression of Jaw1. Thus, more frequent Ca^2+^ peaks brought by the expression of Jaw1 increase the residential time of Ca^2+^ in the cytosol at single cell level. These data provide us a new hypothesis that the augmentative effect of Jaw1 on the sustained Ca^2+^ influx contributes to downstream response activation in Jaw1 expression-specific cells. To verify this concept, the Ca^2+^ dynamics in taste or tuft cells, expressing Jaw1 abundantly under physiological conditions, should be investigated in future studies.

In the Ca^2+^ influx analysis focusing on each ITPR subtype, Jaw1 expression changed the cytoplasmic Ca^2+^ influx oscillation pattern in R1 and R2 SE cells, but not in R3 SE cells. The ability of ITPR3 to induce continuous cytoplasmic Ca^2+^ influx is reportedly the lowest among the three subtypes^19,32^, which might result in the smaller effect of Jaw1 on the residential time in the R3 SE cells. On the other hand, the Jaw1 expression had more augmentative effects on the maximum amplitude in R3 SE cells. Interestingly, the amino acid sequence homology among the subtypes is approximately 65–70%^33^, and their interactors are different depending on the subtypes^34^. These factors might also contribute to the above-described phenotypic differences among the subtypes, although further analyses would be required to clarify these aspects. Intriguingly, the ITPR subtype expression patterns were tissue- and cell-dependently heterogenous. Jaw1 and ITPR2 are expressed in small intestinal tuft cells, chemosensory cells that detect parasitic infection, Jaw1 and ITPR3 in tongue epithelial taste cells that perceive taste, and Jaw1 and ITPR2/3 in pancreatic acinar cells that secrete digestive enzymes. The non-uniform Jaw1 effect on the Ca^2+^ dynamics in each cell type and Jaw1 depletion in MEF reportedly reduced the Ca^2+^ influx while it increased that in pancreatic acinar cells^13–15^. This heterogeneous Jaw1 effect on each ITPR subtype might explain the non-uniform effect of the Jaw1 depletion on Ca^2+^ dynamics. It would be also important to investigate the biological significance of how the ITPR subtype-specific Ca^2+^ influx, as well as ITPR and Jaw1 expression patterns, are involved in the physiological responses in each tissue or cell in vivo.

## Materials and methods

### Cell culture

Each HEK293 Flp-In T-REx cell line (Invitrogen, #78007) was cultured in DMEM supplemented with 10% fetal bovine serum (SIGMA-Aldrich, #F7524), 5.84 mg/mL l-glutamine (Nacalai Tesque, #16919-42), 100 U/mL penicillin, and 100 µg/mL streptomycin (SIGMA-Aldrich, #P4333). Cells were grown in 5% CO_2_ at 37 °C.

### Cell line generation

For the generation of HEK293 Flp-In T-REx Jaw1 KO cells and ITPRs SE cells using the CRISPR/Cas9 system, HEK293 Flp-In T-REx cells were transfected with pSpCas9-2A-Puro Jaw1 or ITPRs sgRNA using the Screen*Fect*™A *plus* (FIJIFILM Wako Pure Chemical, 299-77103). After incubation for 48 h, the cells were subjected to selection by treatment with 2 µg/mL puromycin (Sigma-Aldrich, #P8833). The cells were then single-colonized by the limiting dilution cloning method in a 96-well plate and expanded. For the verification of the mutated sequence in each cell line by Sanger sequencing, genomic DNA was purified from each cell line using QIAamp DNA Micro Kit (QIAGEN, #51304), and the amplified PCR products containing the target region were submitted to the DNA sequence contract service (Genewiz). Two clones for each cell line (Jaw1 KO #7 and #17, R1 SE #11 and #17, R2 SE #5 and #9, and R3 SE #5 and #7) from the cells with mutated sequences were randomly selected and used for following assay to remove the bias behind genetic background between clones. The numbers with cell name are original experimental clone number. Of note, there was no differences in the results between two clones. For the HEK293 Flp-In T-REx Jaw1 IE and Δcoil cell generation, HEK293 Flp-In T-REx Jaw1 KO #17 cells were transfected with pcDNA5 FRT/TO Hu SJaw1 or pcDNA5 FRT/TO Hu SJaw1 Δcoil using Screen*Fect*™A *plus*. After incubation for 48 h, the cells were selected by treatment with 100 µg/mL Hygromycin B (Nacalai Tesque, #07296-66) and expanded.

### Anti-Jaw1 N-ter antibody production

Rabbit polyclonal anti-human Jaw1 antibody was raised against truncated Jaw1 encoding amino acids 1–440 (Operon Biotech). The sequence of human Jaw1 was referred from NCBI (NP_00191055). The serum was subjected to affinity chromatography using N-Hydroxysuccunumidyl-Sepharose 4 Fast Flow (Sigma-Aldrich, #H8280) coupled with the N-terminal region of Jaw1 encoding amino acids1–144, resulting in purified anti-Jaw1 N-ter antibody.

### Plasmids

For the production of pcDNA5 FRT/TO Hu SJaw1, pcDNA3.1 (+) Hu SJaw1 (previously described^35^) was digested with *Bam*HI and *Xho*I, and the fragment was subcloned into a pcDNA5 FRT/TO vector digested with the same enzymes using a commercial DNA ligation kit (TaKaRa, #6023). For the production of pcDNA5 FRT/TO Hu SJaw1 Δcoil, pcDNA3.1 (+) Hu LJaw1 (previously described^35^) was first performed by inverse PCR with primer set 1 resulting in pcDNA3.1 (+) Hu LJaw1 Δcoil. pcDNA3.1 (+) Hu LJaw1 Δcoil was then digested with *Bam*HI and *Xho*I, and the fragment was subcloned into a pcDNA5 FRT/TO vector (digested with the same enzymes) using the aforementioned DNA ligation kit, resulting in pcDNA5 FRT/TO Hu LJaw1 Δcoil. Furthermore, pcDNA5 FRT/TO Hu LJaw1 Δcoil was digested with *Hpa*I and *Xho*I, and the fragment was subcloned into pcDNA5 FRT/TO Hu SJaw1 digested with the same enzymes to swap the N-terminal region of Hu LJaw1 to that of Hu SJaw1.

For the production of pSpCas9-2A-Puro Jaw1 or ITPRs sgRNA, the DNA cassettes containing the sgRNA sequence targeting Jaw1 and the ITPRs were inserted into a pSpCas9(BB)-2A-Puro (PX459) vector (Addgene, #48139) digested with *Bbs*I using the aforementioned DNA ligation kit. Each DNA cassette was produced using primer sets 2–5. The sgRNA targeting ITPRs were designed according to a previous report^18^.

### Western blotting

Each cell line was seeded onto six-well plates (TrueLine, #TR5000) and incubated overnight. The cells were then treated for an additional 24 h with each Dox concentration to induce Jaw1 expression. After that, the cells were harvested by gentle pipetting, washed once with PBS, and lysed with RIPA buffer (50 mM Tris-HCl pH 7.4, 150 mM NaCl, 0.5 mM EDTA, 0.5% NP-40, 0.5% sodium deoxycholate, and 0.1% SDS) containing a protease inhibitor cocktail (Nacalai Tesque, #04080-24). The samples were sonicated for 10 min on ice and centrifuged at 12,000 × *g* for 30 min. The supernatants were added to SDS-PAGE sample buffer and heated at 95 °C for 5 min. The samples were loaded onto a poly acrylamide gel, electrophoresed, and transferred onto polyvinylidene difluoride membranes (GE Healthcare, #10600029). The membranes were blocked with tris-buffered saline containing 0.1% Tween20 (TBS-T) and 3% skim milk (FIJIFILM Wako Pure Chemical, #190-12865) for 1 h at room temperature and incubated overnight at 4 °C with the following primary antibodies: rabbit anti-Jaw1 N-ter (1:1000, produced in our lab), rabbit anti-ITPR1 (1:1000, ABclonal, #A7905), mouse anti-ITPR2 (1:1000, Santa Cruz Biotechnology, #sc-398434), mouse anti-ITPR3 (1:1000, BD Bioscience, #610312), mouse anti-α-Tubulin (1:1000, FUJIFILM WAKO Pure Chemical, #013-25033), or mouse anti-GAPDH (1:1000, FUJIFILM WAKO Pure Chemical, #016-25523). The membranes were washed with TBS-T three times and incubated for 3 h with the following secondary antibodies: anti-Mouse IgG, HRP-Linked Whole Ab Sheep (1:5000, GE Healthcare, #NA931) or anti-Rabbit IgG, HRP-Linked Whole Ab Donkey (1:5000, GE Healthcare, NA934). The membranes were washed with TBS-T three times and reacted with Immuno-star Zeta (FIJIFILM Wako Pure Chemical, #297-72403) or SuperSignal™ West Atto Ultimate Sensitivity Substrate (Thermo Fisher Scientific, #A38554). The bands were detected using an LAS4000 imaging system. The membranes were cut prior to hybridization with antibodies as shown in Supplementary Fig. S4–S13 online, thereby the full-length blots were not prepared.

### Immunostaining

Each cell line was seeded onto 96-well plates (TrueLine, TR5003) and incubated overnight. The cells were treated with each Dox concentration to induce Jaw1 expression. After incubation for 24 h, the cells were fixed with 4% PFA in PBS buffer for 15 min. The cells were then washed with PBS once and permeabilized with 0.2% Triton-X (FIJIFILM Wako Pure Chemical, #162-24755) in PBS for 10 min. Next, the cells were washed with PBS three times and blocked with 2% bovine serum albumin in PBS for 1 h. The cells were then incubated with the following primary antibodies: rabbit anti-Jaw1 N-ter (1:200, produced in our lab) and rat anti-Jaw1 coil (1:200, produced in our lab^16^). After the incubation for 1 h, the cells were washed with PBS three times and incubated for 1 h with Hoechst 33342 along with the following secondary antibodies: goat anti-rat IgG conjugated with Alexa Fluor 488 (1:500, Thermo Fisher Scientific, #A-11006) and goat anti-rabbit IgG conjugated with Alexa Fluor 568 (1:500, Thermo Fisher Scientific, #A-11011). The cells were then washed with PBS three times and observed using fluorescence microscopy (Leica, AF6000-DMI6B).

### Co-immunoprecipitation

Each cell line was cultured on a six-well plate and incubated overnight. The cells were treated with 200 ng/mL Dox to induce Jaw1 expression. After incubation for 24 h, the cells were collected by gentle pipetting and centrifuged at 500 × *g* for 10 min at 4 °C. The pellets were lysed in a lysis buffer (50 mM Tris-HCl, pH 7.6, 150 mM NaCl) containing 0.5% NP-40 and 1 µL of a protease inhibitor cocktail (Nacalai Tesque, 04080-24). The lysates were sonicated for 10 min on ice and centrifuged at 12,000 × *g* for 30 min at 4 °C. The lysates were diluted with lysis buffer into 0.1% NP-40, then incubated with anti-Jaw1 antibody-conjugated beads for 1 h at 4 °C. The conjugated beads were prepared by mixing the anti-Jaw1 N-ter antibody and Protein A Mag Sepharose (Cytiva, #28944006) for 1 h at 4 °C. The beads reacted with the cell lysate were then collected by a magnetic stand and washed with lysis buffer three times. For elution, the beads were mixed with SDS-PAGE buffer and heated at 95 °C for 5 min. On the magnetic stand, the supernatants were collected and subjected to western blotting.

### RT-qPCR

Each cell line was cultured on a six-well plate and incubated overnight. The cells were treated with 200 ng/mL Dox to induce Jaw1 expression. After incubation for 24 h, total RNA was isolated using RNAiso Plus (TaKaRa, 9108). Total RNA was then reverse transcribed using PrimeScript RT Master Mix (TaKaRa, RR036A), and subsequent PCR was performed using TB Green Premix EX Taq II (Tli RNaseH Plus, TaKaRa, RR820A) on Thermal Cycler Dice Real Time System II MRX (TP960, TaKaRa). For *jaw1* and *gapdh* gene detection, primer sets 6 and 7 were used, respectively.

### Calcium assay

Each cell line was seeded onto 96-well black wall plates (Greiner Bio-One, #655090) and incubated overnight. The cells were treated with each Dox concentration to induce Jaw1 expression. For the calcium assay with extracellular Ca^2+^ condition, the cells were incubated for 24 h, washed with PBS once, and incubated with recording buffer (20 mM HEPES, pH 7.4, 115 mM NaCl, 5.4 mM KCl, 1.8 mM CaCl_2_, 0.8 mM MgCl_2_, 13.8 mM _D_-Glucose, and 1.25 mM probenecid) containing 2 µM Fluo-4 AM (dojindo, #F311) or 2 µM Rhod-2 AM (dojindo, #R-002) for 30 min. The cells were then washed with PBS once and incubated with HBSS containing 1.25 mM probenecid for 30 min. For the well scanning calcium assay using a plate reader, Varioskan LUX (Thermo Fisher Scientific) was used. The fluorescence values were recorded every second, and ATP (ORIENTAL YEAST Co., LTD., 45142000) or carbachol (Sigma-Aldrich, C4382) solution was added at 10 s. The data was measured from three independent wells. For the calcium imaging, we used a fluorescent microscope (Leica, AF6000-DMI6B) equipped with a 20X fluor objective. The fluorescent images were captured every 2 s, and ATP solution was added at 10 s. The data was measured from four independent wells. For the calcium assay under Ca^2+^ free conditions, the cells were incubated with Ca^2+^ free recording buffer containing Fluo-4 AM for 30 min, washed with PBS once and incubated with Ca^2+^ free recording buffer. For the well scanning calcium assay for SOCE activity quantification, a plate reader was used and 2 µM thapsigargin solution was added at 10 s and recording buffer with 2 mM CaCl_2_ at 480 s. For the calcium imaging under Ca^2+^ free conditions, fluorescence microscopy was used and the 100 µM ATP solution without Ca^2+^ was added at 10 s.

### Relative Fluo-4 fluorescence intensity quantification

The fluorescence intensity at 0 s was defined as F_0_ and the relative fluorescent intensity at each time point was calculated as F/F_0_. The calculated data was then graphed in GraphPad Prism7.

For the calcium imaging, fluorescent images of the Fluo-4-loaded cells upon ATP stimulation were analyzed and quantified by Fiji. The calcium imaging was performed in the four independent wells and data from 50 cells in each image was used for the quantification. We randomly chose 50 cells from each image, and the center of each cell (8 × 8 pixel) was selected as a representative area. The fluorescent intensity in the chosen area was measured over time for 5 min.

### Ca^2+^ influx type classification

Relative Fluo-4 intensity of each cell was plotted for 5 min using GraphPad Prism 7. The relative Fluo-4 intensity curves were then transferred to layouts and manually classified into four Ca^2+^ influx types as follows: (1) The first group (single type) included the cells with amplitudes only once for 5 min. (2) The second group (oscillation type) included the cells with more than two oscillations. The oscillation was defined as the relative Fluo-4 intensity decreasing below 125% with a peak that rose again during 5 min. (3) The third group (steady reduction type) included the cells that maintained the relative Fluo-4 intensity higher than 125% of the initial values for 5 min. (4) The fourth group (no response type) included the cells maintaining the amplitude less than 300% of the initial value for 5 min. To calculate the ratio, we used 50 cells for each of the four measurements (total of 200 cells).

### Statistical analysis

All statistical tests were calculated using GraphPad Prism 7, and the data are represented as the mean ± S.D. The sample numbers are included in each figure legend. Unpaired, nonparametric Student’s *t*-test was used to compare two sample groups. To compare more than two groups, one-way ANOVA followed by Tukey–Kramer’s *t*-test was used. *, **, ***, and **** indicate statistically significant *P* values of *p* < 0.05, < 0.01, < 0.001, and < 0.0001, respectively. n.s.: non-significant.

## Supplementary Information

Supplementary Information 1.

Supplementary Information 2.

## Data Availability

The data generated or analyzed during this study are included in this published article (see Source data, online).
