# Supplementary material for: Jaw1/LRMP increases Ca2+ influx upon GPCR stimulation with heterogeneous effect on the activity of each ITPR subtype
Source: Sci Rep. 2022 Jun 8;12:9476. doi: 10.1038/s41598-022-13620-4 (PMC9177832; doi:10.1038/s41598-022-13620-4)
Supplement: Supplementary file 2 — Supplementary Information 2. [file 41598_2022_13620_MOESM2_ESM.pdf]

Supplementary Information for:

Jaw1/LRMP increases Ca^{2+} influx upon GPCR stimulation with heterogeneous effect on the activity of each ITPR subtype

Wataru Okumura[1], Takuma Kozono[2], Hiroyuki Sato[3], Hitomi Matsui[4], Tsubasa Takagi[4], Takashi Tonozuka[4], and Atsushi Nishikawa[1,2,3,4*]

[1]Department of Food and Energy Systems Science, Graduate School of Bio-Applications and Systems Engineering, Tokyo University of Agriculture and Technology, Tokyo 184-8588, Japan.
[2]Institute of Global Innovation Research, Tokyo University of Agriculture and Technology, Tokyo 183-8509, Japan.
[3]Cooperative Major in Advanced Health Science, Tokyo University of Agriculture and Technology, Tokyo 184-8588, Japan.
[4]Department of Applied Biological Chemistry, Graduate School of Agriculture, Tokyo University of Agriculture and Technology, Tokyo 183-8509, Japan.

*corresponding. nishikaw@cc.tuat.ac.jp

Supplementary Figure S1

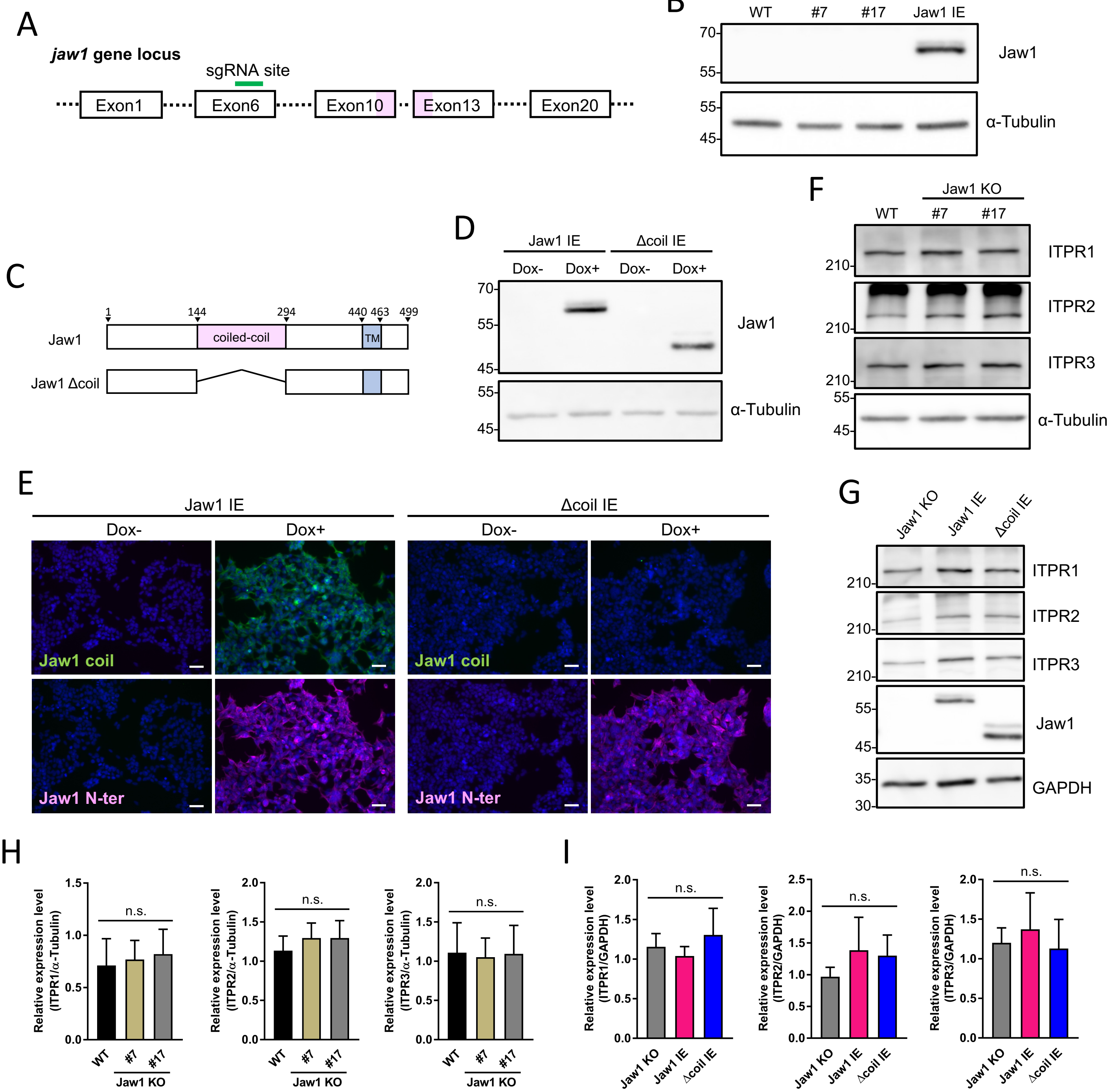

Supplementary Figure S1. Jaw1 depletion or expression do not alter the ITPR expression levels. (**A**) Schematic representation of the CRISPR/Cas9 sgRNA targeting site in the *jaw1* gene locus. The sgRNA was designed to target the region upstream of the coiled-coil domain (shaded with pink). (**B**) WT, Jaw1 KO #7, Jaw1 KO #17, and Jaw1 IE cells subjected to western blotting to evaluate the endogenous Jaw1 expression in the WT cells. Images used for western blots are shown in Supplementary Fig. S7A,B online. (**C**) Schematic representation of human Jaw1 and Jaw1 Δcoil. coiled-coil, coiled-coil domain; TM, transmembrane domain. (**D**,**E**) Jaw1 IE and Δcoil IE cells cultured with (Dox+) or without (Dox−) 200 ng/mL of Dox and subjected to western blotting (**D**) or immunostaining using Hoechst33342 (blue), anti-Jaw1 coil antibody (green), and anti-Jaw1 N-ter antibody (magenta). Images used for western blots are shown in Supplementary Fig. S8A,B, online. (**E**). Scale bars: 10 μm. (**F**) WT, Jaw1 KO #7 and Jaw1 KO #17 cells subjected to western blotting to evaluate the ITPR expression levels. Images used for western blots are shown in Supplementary Fig. S9A-D, online. (**G**) Jaw1 KO, Jaw1 IE, and Δcoil IE cells treated with 200 ng/mL of Dox for 24 h and subjected to western blotting to evaluate the Jaw1 and ITPR expression levels. Images used for western blots are shown in Supplementary Fig. S10A-E, online. (**H**,**I**) Graph representing the relative ITPR1, ITPR2, and ITPR3 expression levels with α-Tubulin in (**F**) and (**H**) and GAPDH in (**G**) and (**I**). n = 3. The error bar shows ±S.D.; n.s., non-significant. Tukey–Kramer *t*-test.

Supplementary Figure S2

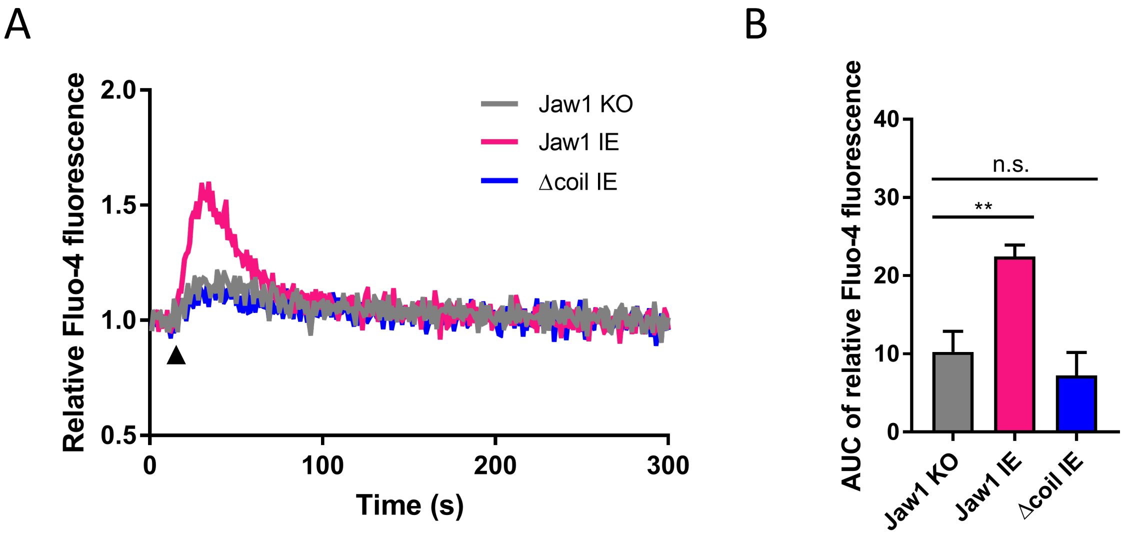

Supplementary Figure S2. Jaw1 increases the Ca^{2+} influx upon carbachol stimulation. (**A**) Mean curves of relative Fluo-4 intensity upon 100 μM carbachol stimulation measured using a plate reader. The closed triangles indicate the time point of 100 μM carbachol solution supplementation. (**B**) AUC (0-100 s) in (**A**). n = 3. The error bar shows ±S.D.; n.s., non-significant; **, p < 0.01, Tukey–Kramer's *t*-test.

Supplementary Figure S3

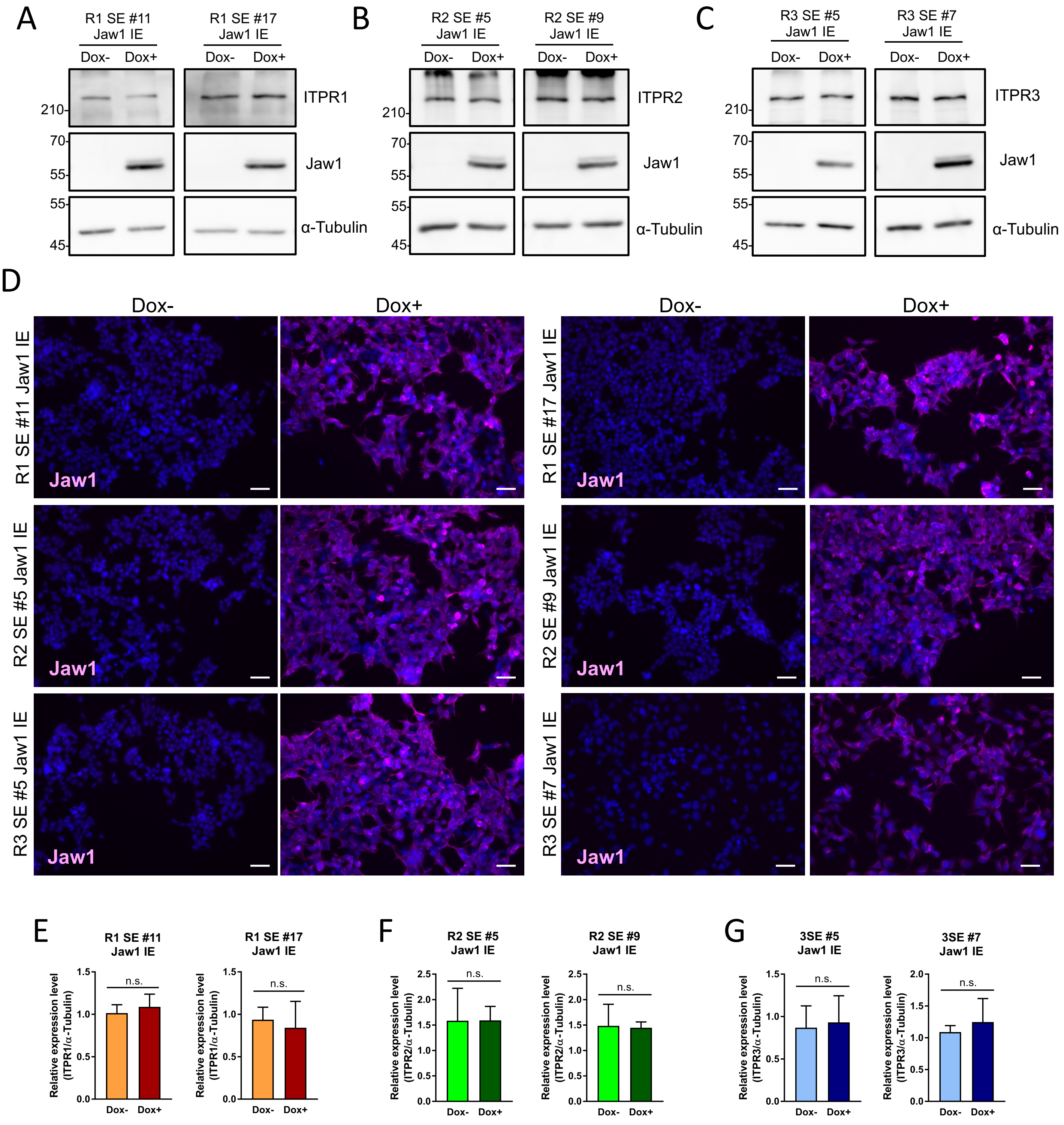

Supplementary Figure S3. The ITPR expression levels are not altered in ITPRs SE cells. (**A**,**B**,**C**,**G**) R1 SE #11 Jaw1 IE or R1 SE #17 Jaw1 IE cells (**A**), R2 SE #5 Jaw1 IE or R2 SE #9 Jaw1 IE cells (**B**) or R3 SE #5 Jaw1 IE or R3 SE #7 Jaw1 IE cells (**C**) cultured with (Dox+) or without (Dox−) 200 ng/mL of Dox and subjected to western blotting (**A**,**B**,**C**) or immunostaining using with Hoechst33342 (blue) and anti-Jaw1 N-ter (magenta). Images used for western blots are shown in Supplementary Fig. S11-S13, online. (**G**). Scale bars: 10 µm. (**D**,**E**,**F**) Graph representing the relative ITPR1, ITPR2 and ITPR3 expression levels (**A**,**B**,**C**), respectively, with α-Tubulin. n = 3. The error bar shows ±S.D.; n.s., non-significant. Student's *t*-test.

Supplementary Figure S4

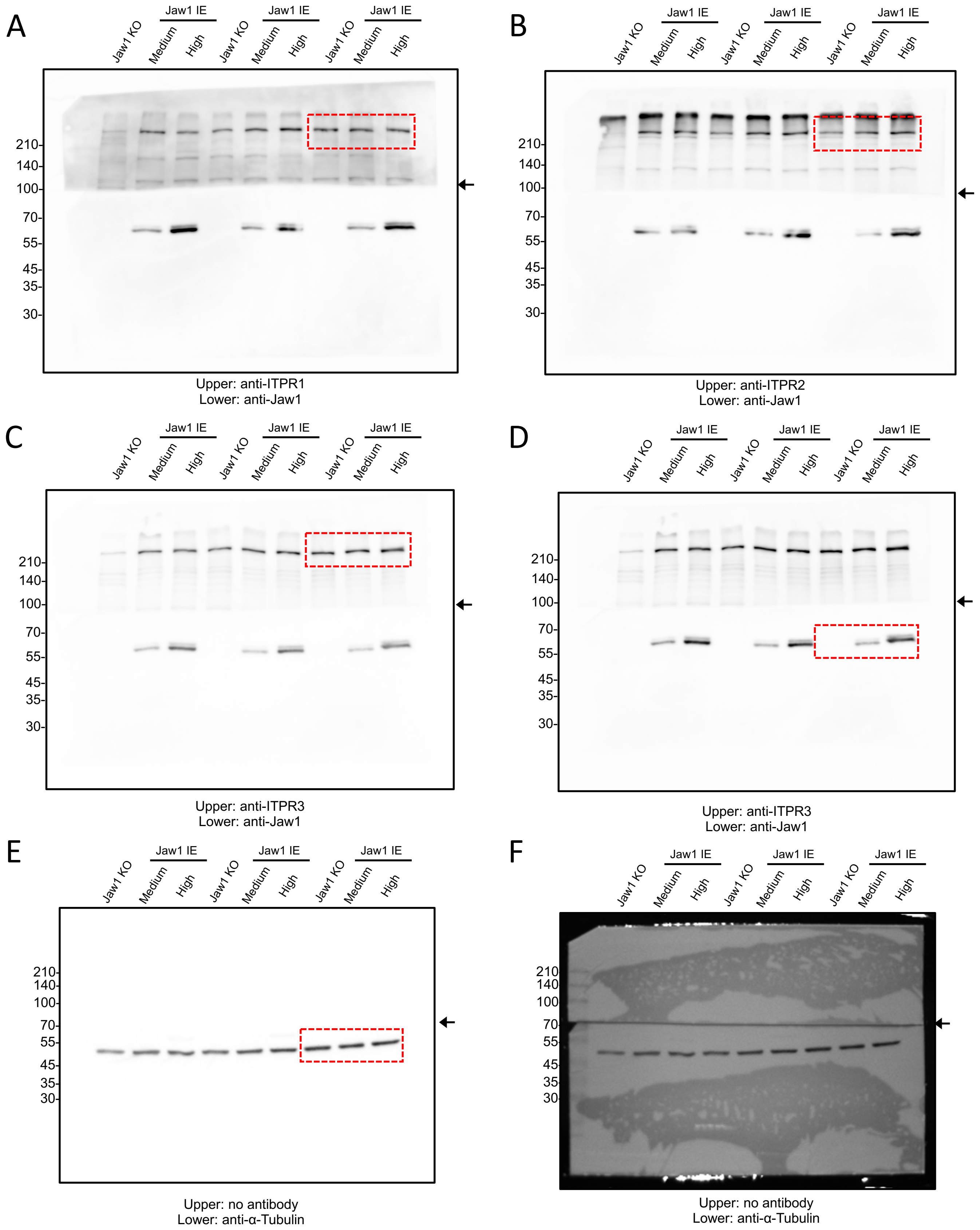

Supplementary Figure S4. Images used for western blots of Fig. 1J. The membranes were divided at the black arrow point and incubated with indicated antibodies. The blots of (A-E) were cropped at the red broken line square area and summarized in Fig. 1J. The blot of (F) is the merged image of (E) and the bright field since the blot of (E) is too high contrast to see the edge of the membrane.

Supplementary Figure S5

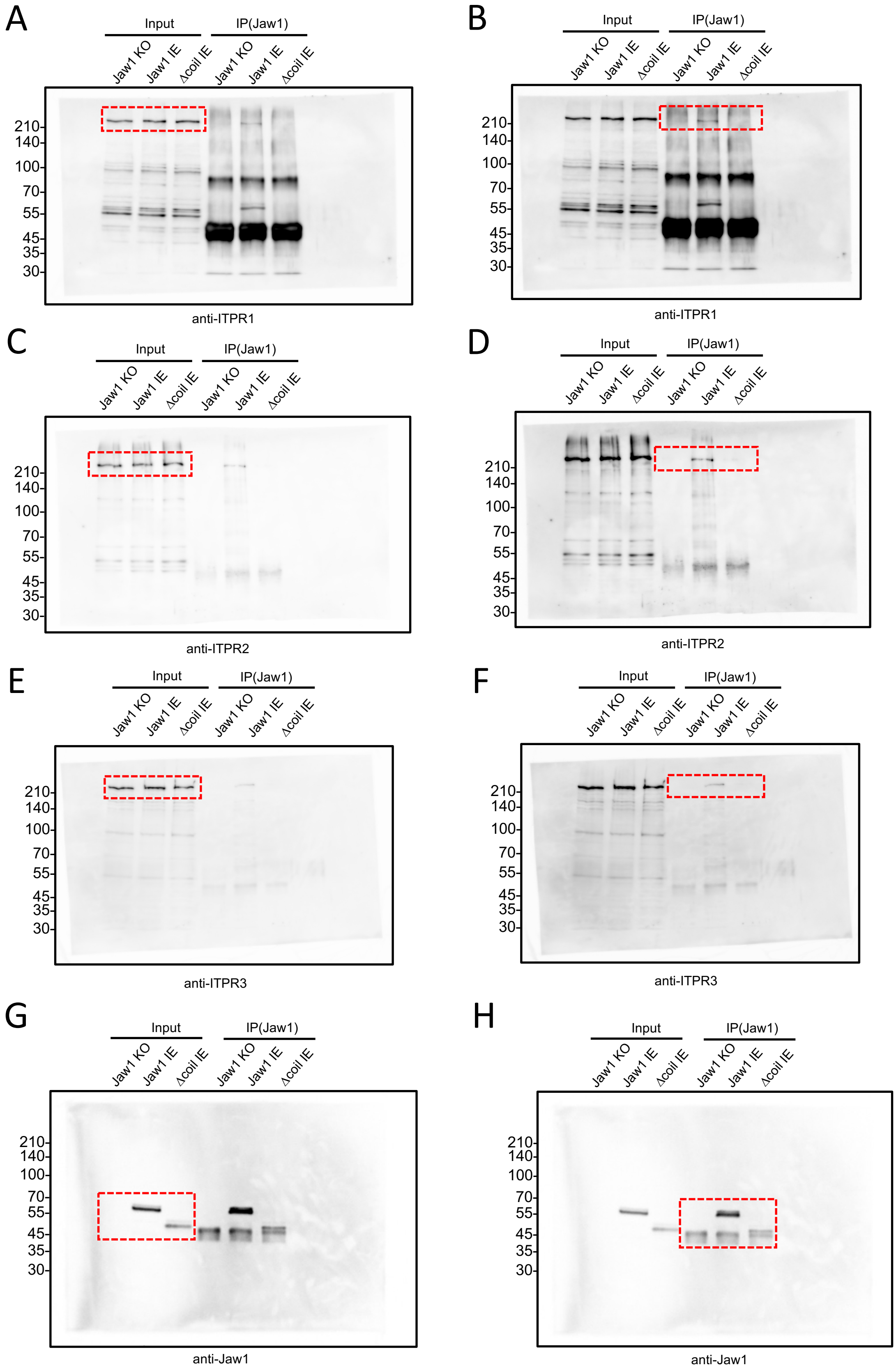

Supplementary Figure S5. Images used for western blots of Fig. 5A. The membranes were incubated with indicated antibodies. The blots of (A-H) were cropped at the red broken line square area and summarized in Fig. 5A.

Supplementary Figure S6

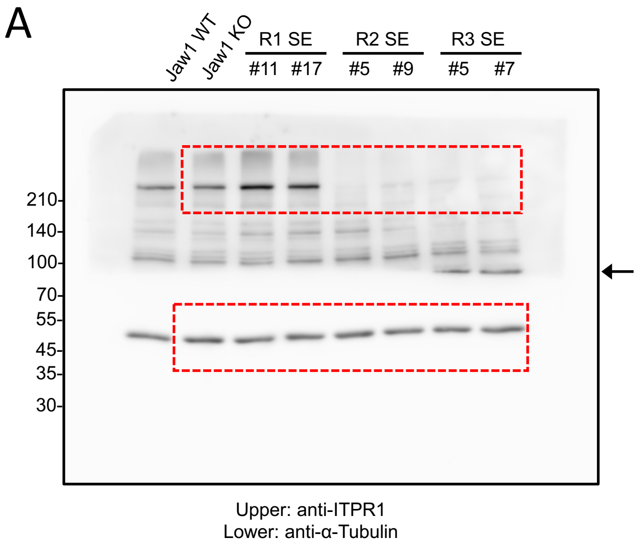

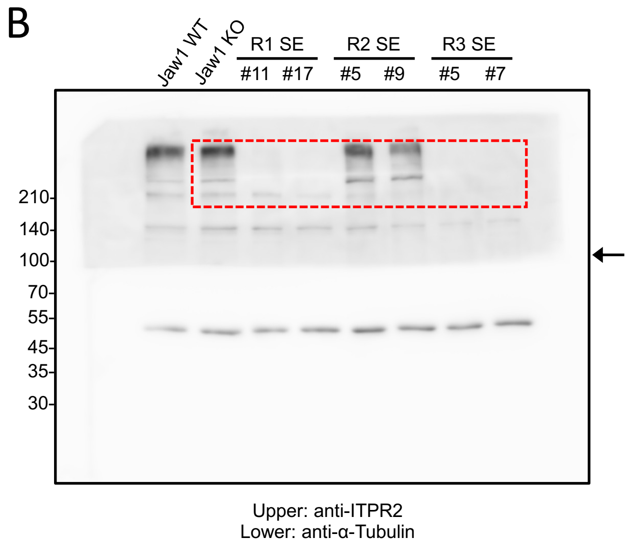

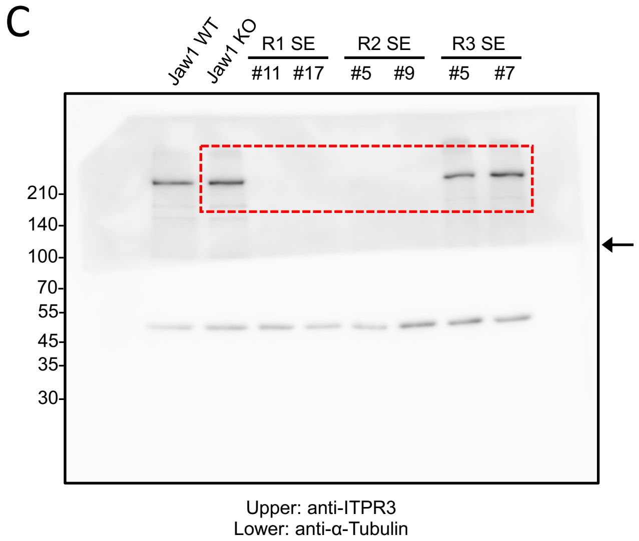

Supplementary Figure S6. Images used for western blots of Fig. 5B. The membranes were divided at the black arrow point and incubated with indicated antibodies. The blots of (A-C) were cropped at the red broken line square area and summarized in Fig. 5B.

Supplementary Figure S7

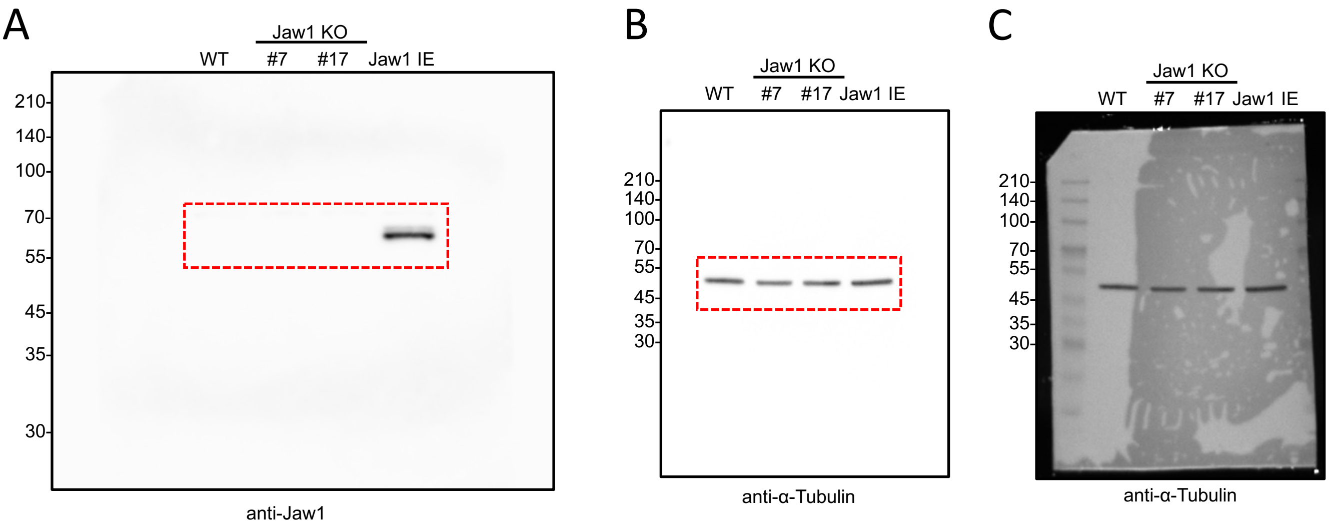

Supplementary Figure S7. Images used for western blots of Fig. S1B. The membranes were incubated with indicated antibodies. The blots of (A, B) were cropped at the red broken line square area and summarized in Fig. S1B. The blot of (C) is the merged image of (B) and the bright field since the blot of (B) is too high contrast to see the edge of the membrane.

Supplementary Figure S8

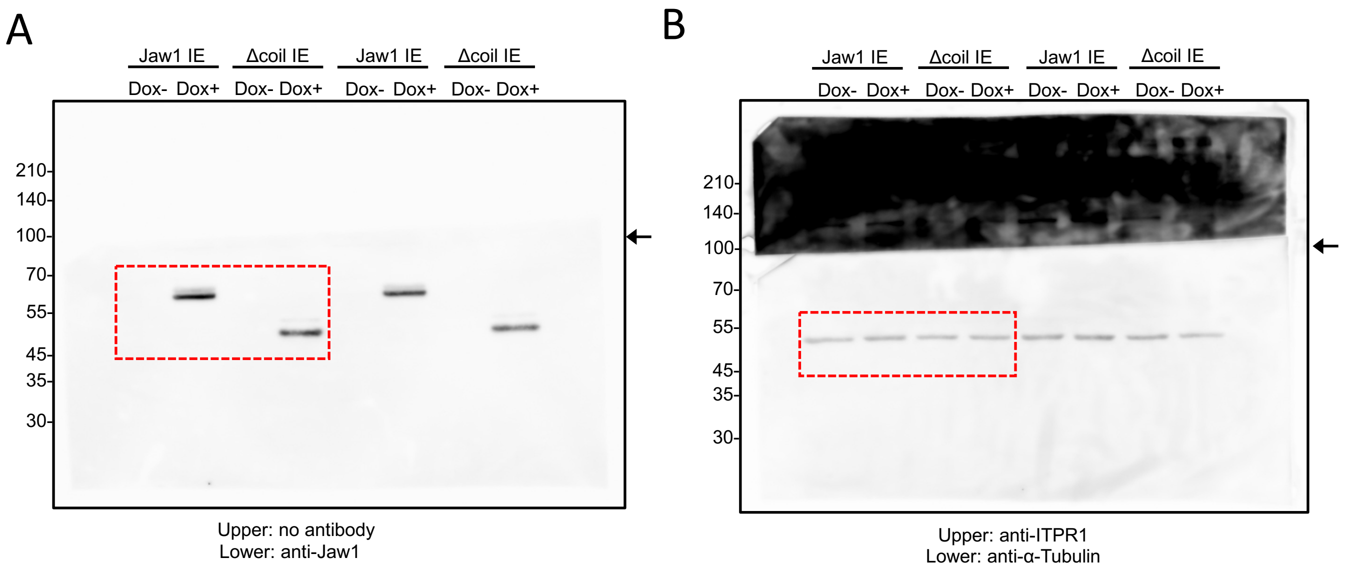

Supplementary Figure S8. Images used for western blots of Fig. S1D. The membranes were divided at the black arrow point and incubated with indicated antibodies. The blots of (A, B) were cropped at the red broken line square area and summarized in Fig. S1D.

Supplementary Figure S9

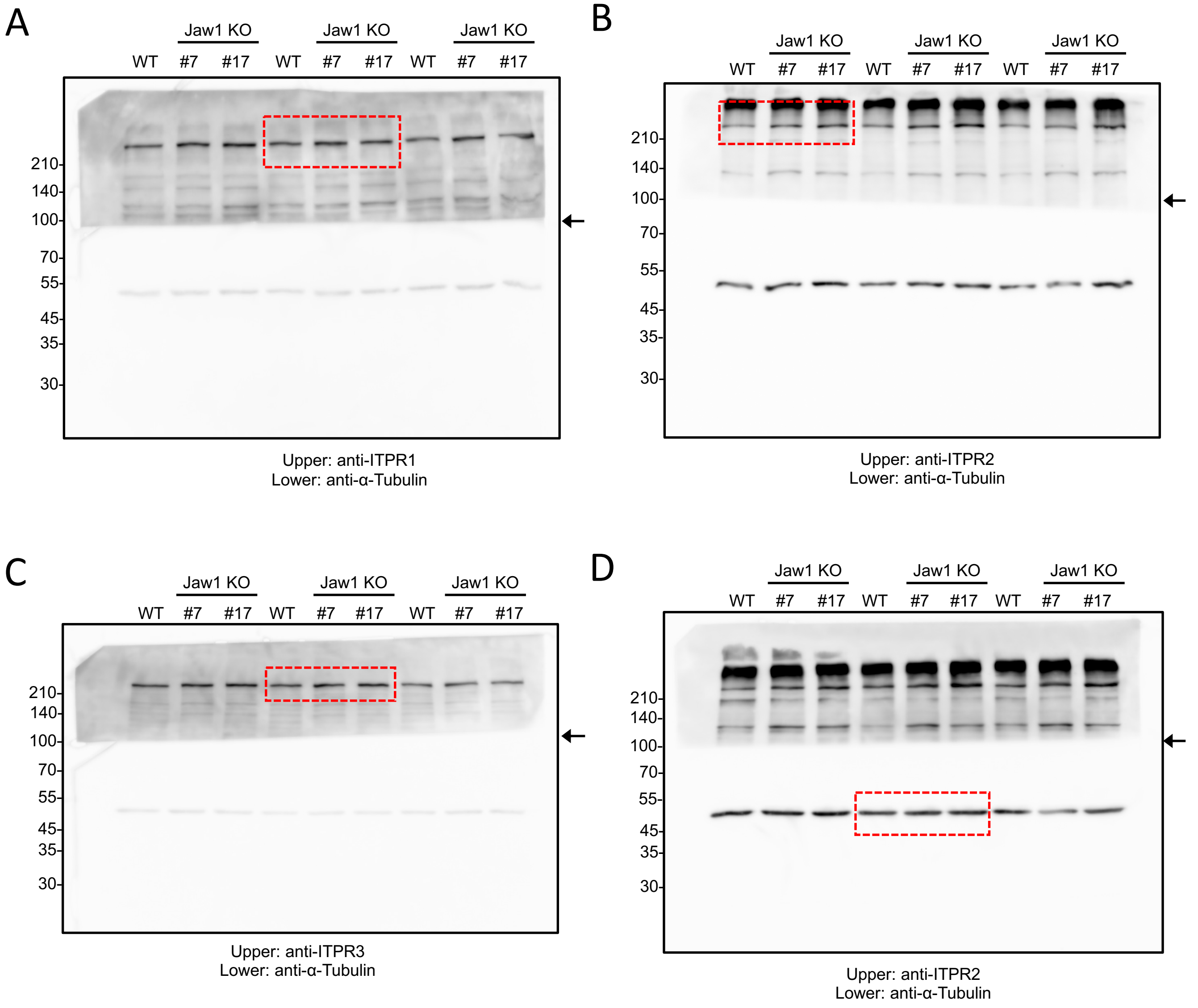

Supplementary Figure S9. Images used for western blots of Fig. S1F. The membranes were divided at the black arrow point and incubated with indicated antibodies. The blots of (A-D) were cropped at the red broken line square area and summarized in Fig. S1F.

Supplementary Figure S10

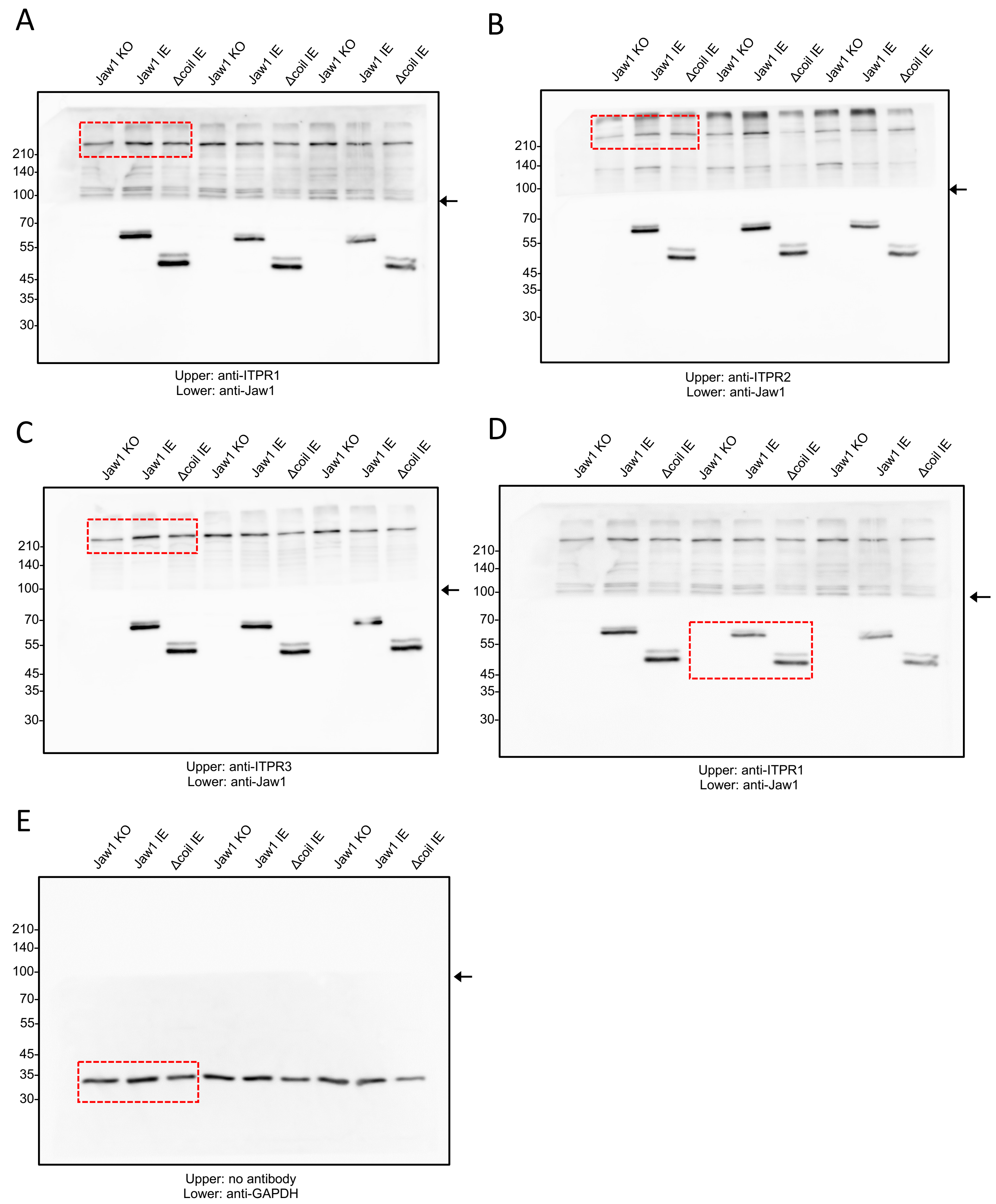

Supplementary Figure S10. Images used for western blots of Fig. S1G. The membranes were divided at the black arrow point and incubated with indicated antibodies. The blots of (A-E) were cropped at the red broken line square area and summarized in Fig. S1G.

Supplementary Figure S11

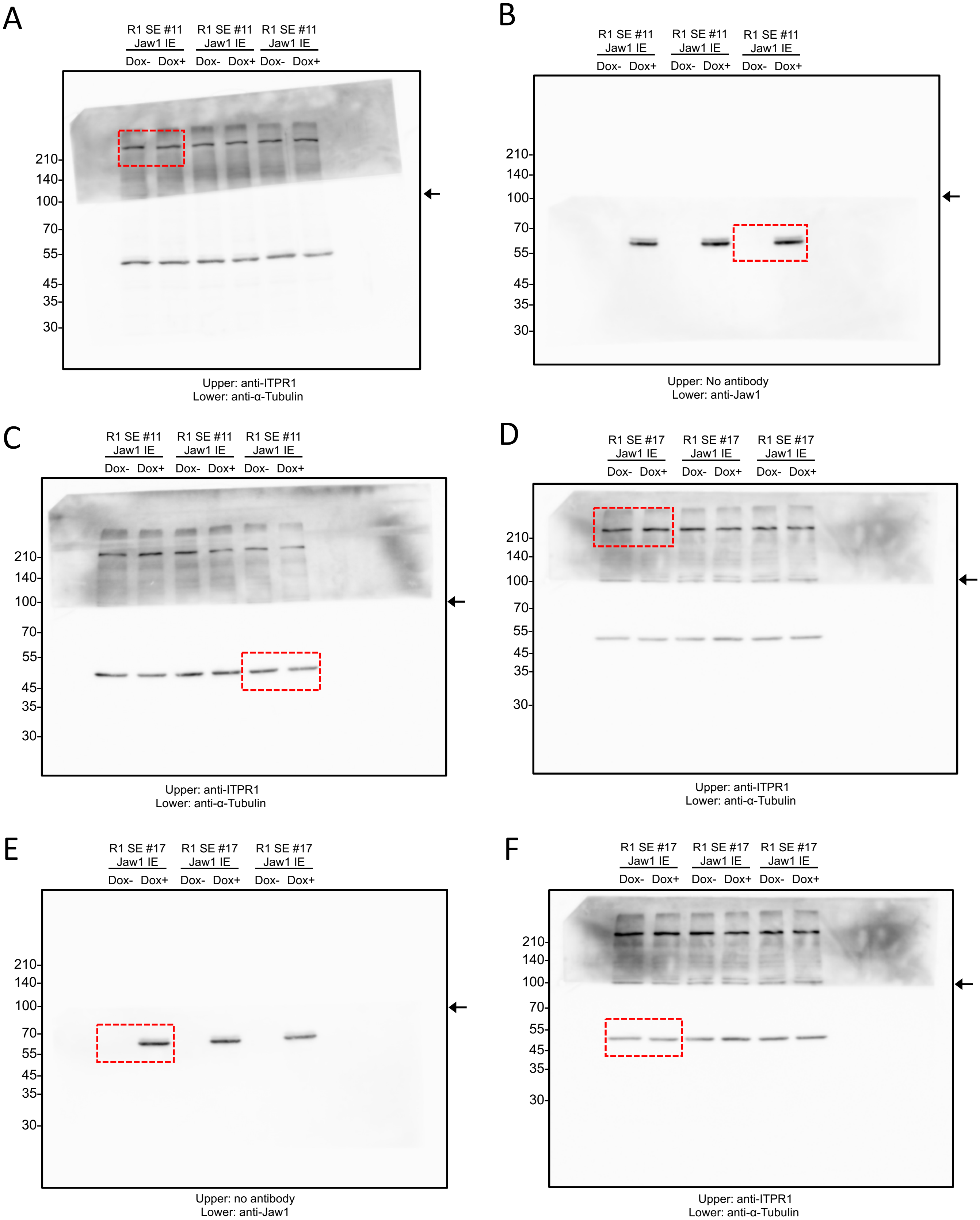

Supplementary Figure S11. Images used for western blots of Fig. S3A. The membranes were divided at the black arrow point and incubated with indicated antibodies. The blots of (A-F) were cropped at the red broken line square area and summarized in Fig. S3A.

Supplementary Figure S12

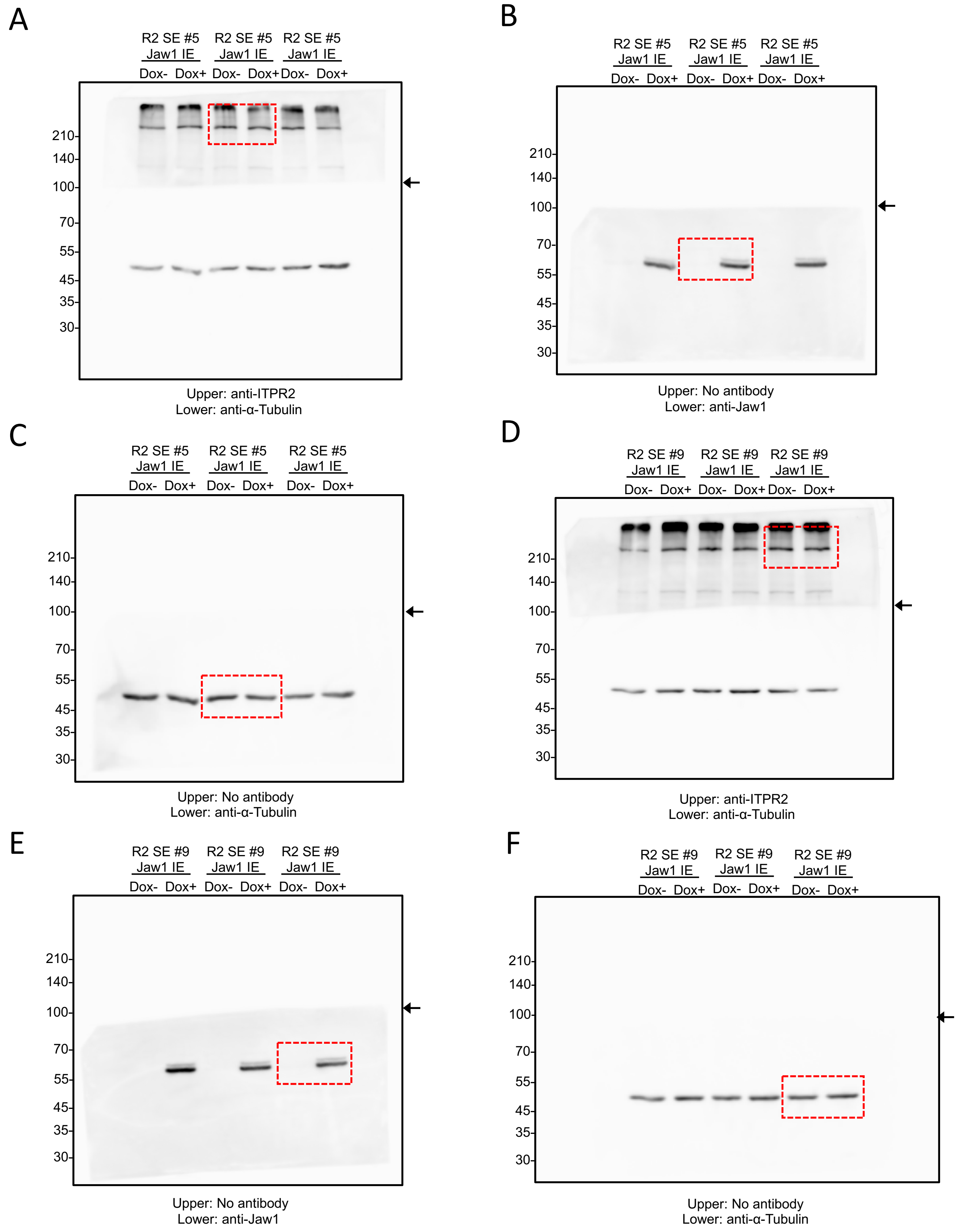

Supplementary Figure S12. Images used for western blots of Fig. S3B. The membranes were divided at the black arrow point and incubated with indicated antibodies. The blots of (A-F) were cropped at the red broken line square area and summarized in Fig. S3B.

Supplementary Figure S13

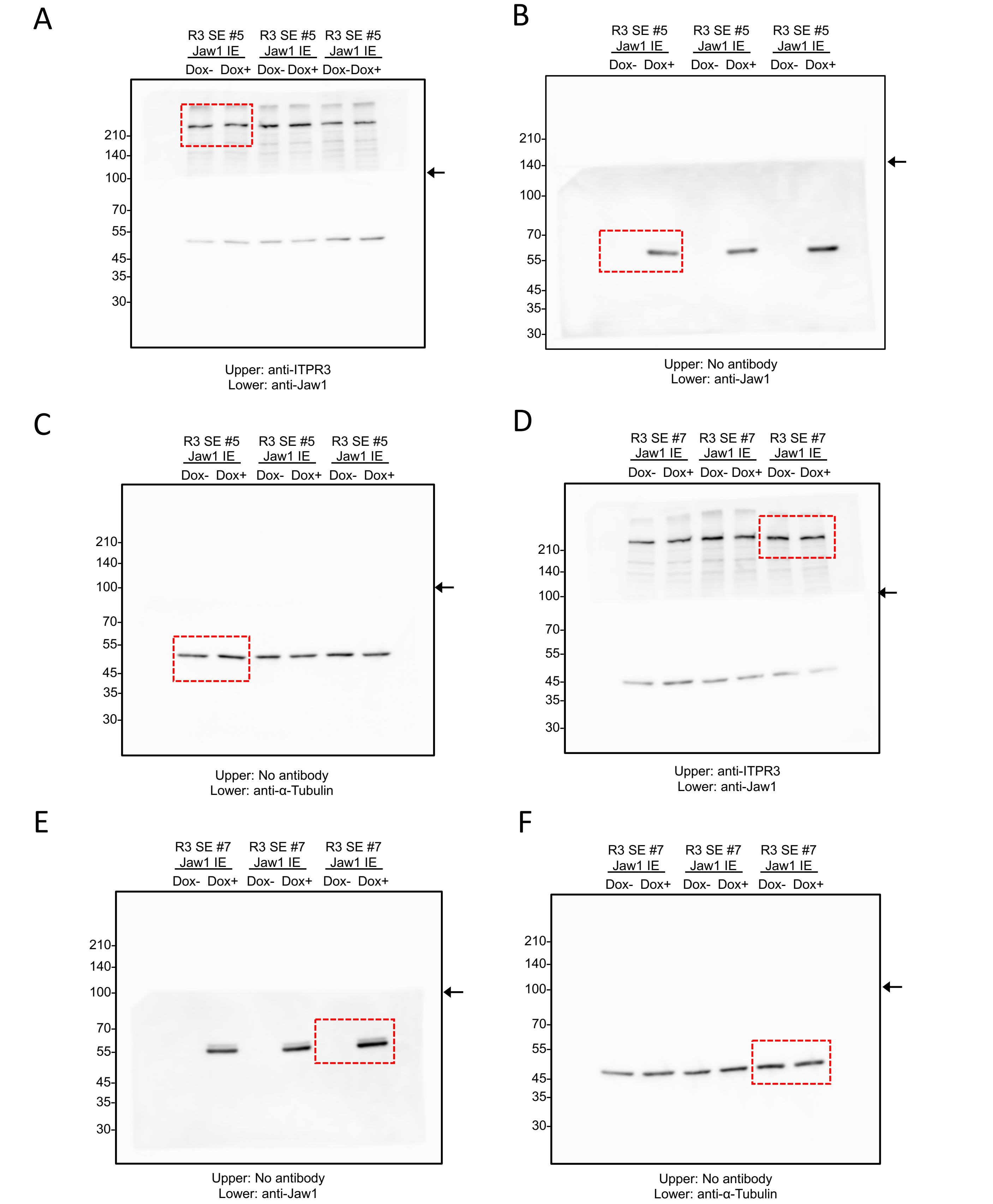

Supplementary Figure S13. Images used for western blots of Fig. S3C. The membranes were divided at the black arrow point and incubated with indicated antibodies. The blots of (A-F) were cropped at the red broken line square area and summarized in Fig. S3C.

Supplementary table S1

Number	Orientation	Sequence
Primer set1	Forward	5′-TTAGACAAGAAGATGATGATGACTGCCAAATTAAAAAAC-3′
	Reverse	5′-TCATCTTCTTGTCTAACGTTAACCGATTTCACAGTG-3′
Primer set 2	Forward	5′-CACCGATCAGCTTCTCCCACGATAG-3′
	Reverse	5′-AAACCTATCGTGGGAGAAGCTGATC-3′
Primer set 3	Forward	5′-CACCGTTTGTTCTCTGTACGCGG-3′
	Reverse	5′-AAACCCGCGTACAGAGAACAAAC-3′
Primer set 4	Forward	5′-CACCGTTTGTTCTCTGTACGCGG-3′
	Reverse	5′-AAACAACAGTTTCTTATTCTCCGAC-3′
Primer set 5	Forward	5′-CACCGGTGCCCCATGAACCGCTACT-3′
	Reverse	5′-AAACAGTAGCGGTTCATGGGGCACC-3′
Primer set 6	Forward	5′-GTGACTGGTTTACCTTGGAG-3′
	Reverse	5′-CTTTATACTCTTCTCCAGCTTCTT-3′
Primer set 7	Forward	5′-GGTGCTAAGCAGTTGGTGGT-3′
	Reverse	5′-GAGTCCACTGGCGTCTTCAC-3′

Supplementary table S1. List of primer sets used in this study.
